# Cervical fibroids: the vaginal intracapsular myomectomy with classification by the fibroids’ origin, growth directions, and localizations

**DOI:** 10.3389/fmed.2025.1564667

**Published:** 2025-05-09

**Authors:** Andrea Tinelli, Ospan Mynbaev, Gaetano Panese, Martina Licchelli, Giovanni Pecorella, Antonio Malvasi, Ioannis Kosmas, Michael Stark, Wenming Cao

**Affiliations:** ^1^Department of Obstetrics and Gynecology and CERICSAL, CEntro di RIcerca Clinico SALentino, Veris Delli Ponti Hospital, Scorrano, Lecce, Italy; ^2^The New European Surgical Academy (NESA), Berlin, Germany; ^3^Department of Interdisciplinary Medicine (DIM), Unit of Obstetrics and Gynecology, University of Bari, Bari, Italy; ^4^Department of Obstetrics and Gynecology, Ioannina State General Hospital G. Hatzikosta, Ioannina University, Ioannina, Greece; ^5^Department of Gynecology, Pingshan District Central Hospital of Shenzhen, Shenzhen, China

**Keywords:** uterine cervix, cervical leiomyoma, cervical fibroids, cervical intracapsular myomectomy, vaginal myomectomy, laparoscopic myomectomy, open myomectomy, laparotomic myomectomy

## Abstract

**Aim of the study:**

To define the impact of cervical fibroids (CFs) localization, size, and their combined presence on the feasibility of performing cervical intracapsular myomectomy (CIME) by using a vaginal approach.

**Objective:**

Evaluation of the feasibility of conducting CIME via the transvaginal approach.

**Materials and methods:**

This retrospective cohort observational study included 32 patients who underwent CIME. The findings were evaluated by comparing them with the outcomes of 1,204 patients with CFs reported in the English and Chinese literature.

**Results:**

The study demonstrated that CIME by vaginal approach can be successfully performed on CFs with a mean size of 8.08 cm (95% CI: 7.44–8.73 cm) for extracervical anterior and posterior sites, 7.61 cm (95% CI: 7.09–8.14 cm) for extracervical and intracervical lateral localizations, and 5.36 cm (95% CI: 4.71–6.35 cm) for combined CFs in two different localization sites. The feasibility and efficiency of the suggested CIME technique were confirmed through a comparative analysis of perioperative endpoints from 326 vaginal, 643 laparoscopic, and 235 laparotomic myomectomies documented in the literature.

**Conclusion:**

The findings highlight key issues related to the suggested original CIME technique performed via a direct vaginal approach. They also provide a comprehensive classification of CFs along with the CFs’ clinical manifestations based on the type of growth direction, locations, and fibroid maturity. These findings may have implications for managing patients with CFs.

## 1 Introduction

The uterine cervix, a vital component of a woman’s health and well-being (1), serves as a bridge between the uterine cavity and the vagina. Its cervical mucus acts as a defense against infections from the lower vaginal tract (1, 2). The cervix plays a crucial role in sexual activity (3), fertilization (2), pregnancy (4), and childbirth (5). Its volume and structure are influenced by age, menstrual cycle, pregnancy, and hormone therapies (4–8).

In nulliparous women, the cervix’s topographic morphology is thinner and cone-shaped, while in parous women, it is generally cylindrical and thicker (5). However, the development of neoplasms, trauma during childbirth, abortions, infections, inflammatory processes, and surgical procedures can all disrupt the cervical shape, architecture, and tissue structure (4–6, 9–14). These factors contribute to the complexity of cervical health, and conditions like cervical fibroids (CFs) cause anatomical distortion with various clinical symptoms (9, 10, 12–20).

The prevalence of CFs in the literature ranges from 0.6 to 7.7% (21–26), and current visualization methods have improved the diagnosis and management of CFs. The increased number of publications with case reports in the worldwide literature described complications in obstetric patients (27–29). It demonstrated the increased size of CFs filled in the pelvic cavity (12, 15, 17, 18, 25, 30), with a specific appearance as the Lantern on Saint Paul’s dome (10) and atypical presentation with mimicking symptoms of pathologic conditions of neighboring organs (15, 19, 20), gastrointestinal, and urologic systems (16–18). Symptomatic CFs could lead to life-threatening conditions during pregnancy, delivery, and postpartum periods (14, 27–29), hamper the daily routine activity of women, and reduce the quality of their lives (12, 16, 17, 27–29).

Therefore, many diagnostic and treatment technologies to manage CFs were described in the literature (23–25, 30–37). According to the recent systematic literature review, laparoscopy (LS) was the most frequently used approach in 114 (59.4%) patients among 192 identified cases of surgical and radiologic interventional treatments (37). Then, laparotomy (LT) was used in 50 (26.0%) cases, interventional radiology in 20 (10.4%) patients, and vaginal myomectomy (VME) only in 8 (4.2%) women. Most papers in this study addressed this topic as case reports, and VME is still a rarely utilized method to treat CFs (37). Because there is a risk of significant bleeding and other complications, as well as injury to the uterine blood vessels and surrounding organs, the removal of CFs through the vagina demands surgical expertise and experience. As a result, these challenges have restricted the use of the direct anatomic surgical approach.

This study attempted to explore the feasibility of surgically removing the CFs through the vagina, depending on fibroids size, their single or combined occurrence, and localization sites in the cervix with further comparative analysis of findings with extensive data from the literature. In addition, based on personal experience and a thorough analysis of the English and Chinese literature, an original classification of CFs will be suggested.

## 2 Materials and methods

### 2.1 Study design and subjects

This retrospective cohort observational study was planned by the EQUATOR Reporting Guidelines, which include the STROBE Statement - Checklist of items (Supplementary Table 1). We used the medical records of patients treated in the Division of Experimental Endoscopic Surgery, Imaging, Technology, and Minimally Invasive Therapy, Lecce, and the Department of Obstetrics and Gynecology and CERICSAL (Centro di RIcerca Clinico SALentino), “Veris Delli Ponti Hospital,” Scorrano, as well as the Department of Obstetrics and Gynecology and CERICSAL (CEntro di RIcerca Clinico SALentino). The local IRB approved our study protocol. Every patient signed an informed consent form granting permission for data collection, analysis, and publication in an anonymous manner (The IRB code of authorization is T7WGSJ3). The creation and dissemination of the intracapsular myomectomy (ME) method, which allows the fibroid to be removed from the uterus inside its pseudocapsule—a fibro-neurovascular network that divides the tumor from the myometrium—was accomplished by our multinational team. It was then thought that this method could start the myometrial muscle’s natural healing process following surgery. This intracapsular myomectomy technique was first applied in open and laparoscopic procedures to treat uterine fibroids. Since 2014, the vaginal method of enucleating CFs has used the application of intracapsular myomectomy. Thus, the term cervical intracapsular myomectomy (CIME) was coined for this novel technique. For this study, we collected anonymous medical records in an Excel table from 32 patients who underwent CIME via the vagina in the 2014–2023-time span and were followed up until January 2024 (Supplementary Table 2). Sixteen patients were operated on between 2014–2020 years, four patients in 2021, seven women in 2022 and five patients in 2023.

**Figure 1:**
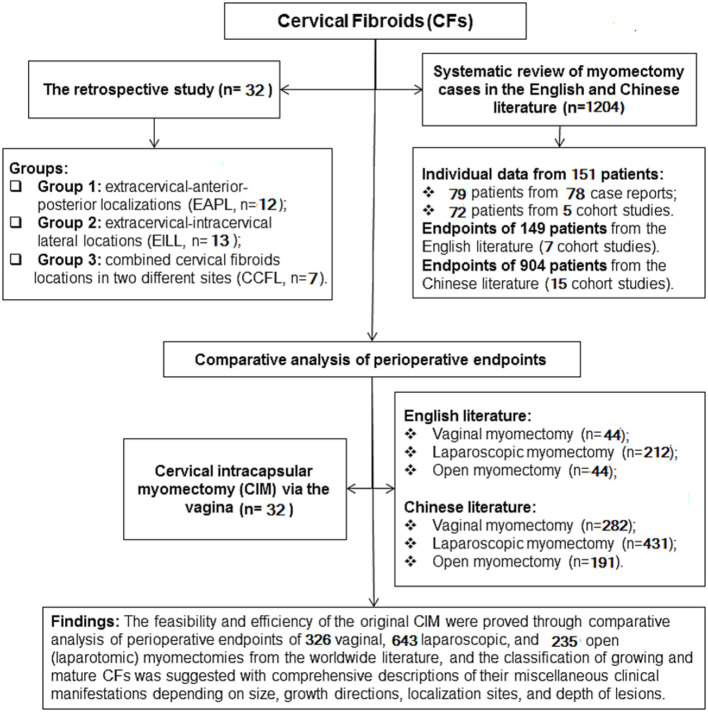
A flowchart of the retro cervical cohort observational study with comprehensive literature review.

These records were the subjects of our retrospective cohort observational study, which included a comprehensive review of the literature in both English and Chinese (Figure 1). This extensive review allowed us to gather more cases of CFs, classify CFs, investigate the feasibility of treating CFs surgically through vagina, and assess the effectiveness and safety of this approach based on the location, size, and occurrence of either a single or a combination of CFs.

### 2.2 Tested hypotheses

The possibility of CIME via the vagina might be depended on the single or combined occurrence of CFs and their localization sites. The size of CFs may impact the performance of surgical technique and its outcome. The localization site of CFs and their single or combined occurrence might affect intrasurgical and postsurgical outcomes.

### 2.3 Patient grouping

Depending on the localization of CFs, thirty-two patients were divided into three groups: Extracervical-anterior–posterior localizations (EAPL, *n* = 12) group 1; extracervical-intracervical lateral localizations (EILL, *n* = 13) group 2; combined cervical fibroid localizations (CCFL, *n* = 7) group 3 (Figure 1).

### 2.4 Demographic, surgical, and postoperative data

The following main demographic and clinical parameters were collected:

- Age;
- Parous condition;
- CF-related symptoms;
- Indications for surgery;
- Number and size of CFs;
- Located positions of CFs;
- Fibroid-attached cervical structure (anterior or posterior lips or lateral sites);
- The growth direction of CFs;
- Surgery time;
- Estimated blood loss;
- Discharge day.

We also registered body mass index (BMI), parity (prior pregnancies and deliveries), hemoglobin (Hb) level before and during the early follow-up, and the remission of symptoms during the short-term follow-up, including general and local complications (bleeding, anemia, local vaginal and peri-cervical inflammatory infections). In addition, we gathered additional surgical, perioperative, and postoperative data, which is of utmost importance in understanding the complete picture of CFs treatment outcomes.

The long-term follow-up included yearly telephone interviews to assess potential fibroid recurrence and pregnancy outcomes, if necessary, clinical exams, and ultrasounds every year for up to three years.

### 2.5 Standardized preoperative preparation of patients

Every patient had regular preoperative examinations, as well as vaginal and blood smear testing. Transvaginal ultrasound (TUS) and magnetic resonance imaging (MRI) diagnosed the location and extent of CFs. Regarding symptomatic CFs, no preoperative therapies were used. Patients were given antibiotic prophylaxis if needed and vaginal flora recovery treatment before surgery. Twelve hours before surgery, on the day of admission, the anesthesiologist assessed the patients and performed an intestinal purge as a routine protocol in our Hospital.

### 2.6 Surgical protocol

**Figure 2:**
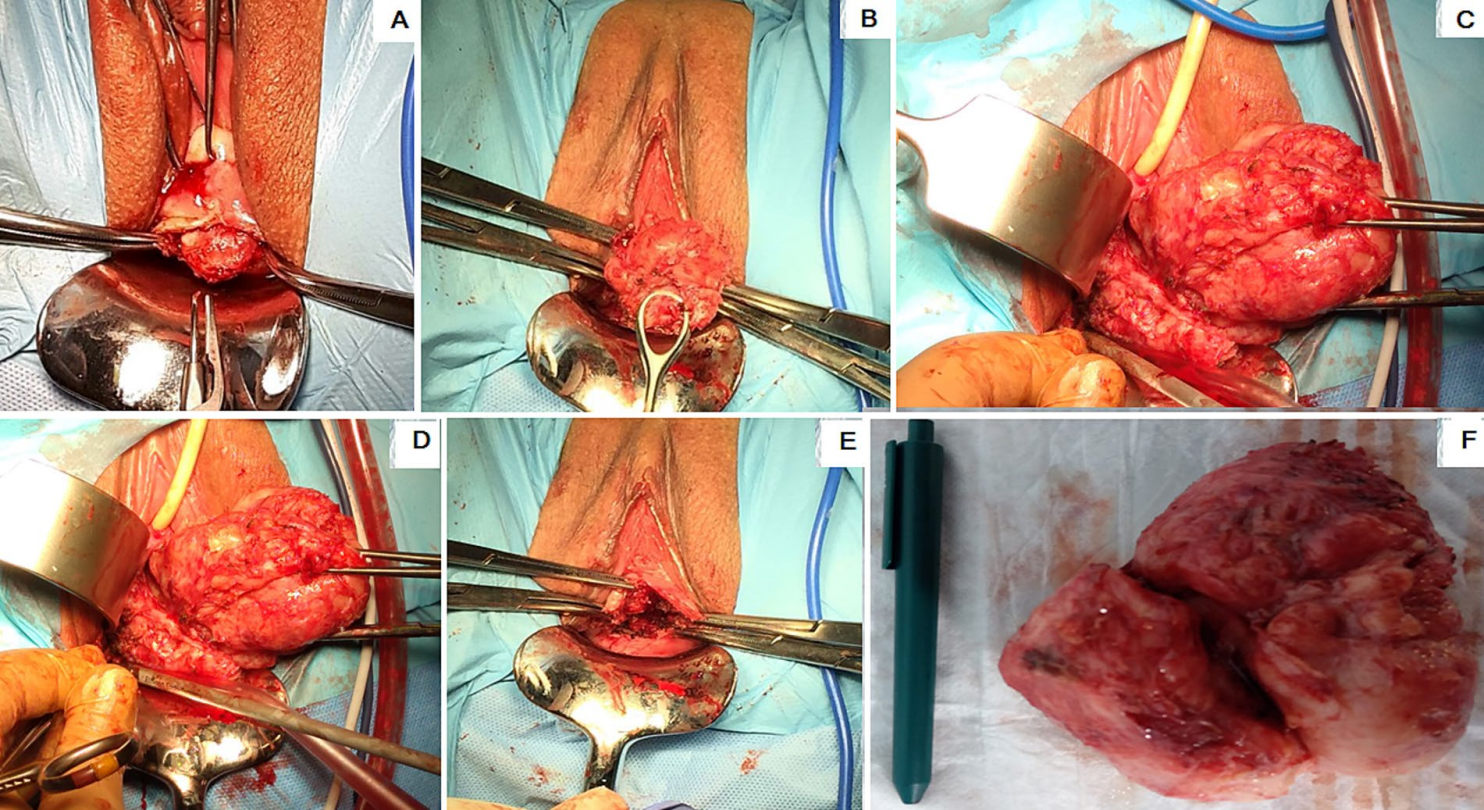
Steps of the cervical intracapsular myomectomy: **(A)** The protrusion of the uterine cervical fibroid (CF) lower portion; **(B)** hooked and pulled CF by the ring forceps; **(C)** coagulation of the CF pseudocapsule fibro-neurovascular fiber; **(D)** the CF hooked and pulled up with Collin’s forceps; **(E)** uterine cervix after CF removal; **(F)** the removed CF placed next to a pen.

Under general anesthesia, surgeries were carried out in the lithotomy positions for the patients. Following disinfection, a sterile drape was placed over the surgical site, and a Foley catheter (Teleflex, USA) was placed inside the bladder. The Collins’ forceps (Integra, USA) clamped the upper and lower cervical lips. The uterine cervix was dragged as far down as possible to exert gentle traction opposite the fibroid. If the fibroid was posterior, it was drawn anteriorly; if it was posterior, it was pulled down. The uterine cervix was later dragged to opposing positions (left or right) in lateral localizations of CFs. The pseudocapsule encircling the fibroid was discovered after the cervical wall above it was cut (Figure 2A). The fibroid was exposed by bipolar scissors or Harmonic Focus Shears with Adaptive Tissue Technology (Ethicon, J&J, Newark, USA). Collins’ forceps grabbed it and gently pulled it toward the vulva (Figure 2B). Subsequently, the procedure followed the standard intracapsular myomectomy protocol, performed by laparotomy and laparoscopy. This technique allowed us to preserve the neurovascular bundles within the fibroid pseudocapsule (Figures 2C,D) performed by selective coagulation. This technique enabled us to successfully remove the fibroid from the cervix (Figures 2E,F). Following hemostasis, single or continuous knots were used to stitch the edges of the wounds using either monofilament or interwoven absorbable sutures 5/0 (Ethicon, J&J, Newark, USA). Finally, the hemostatic check-up was performed at the end of the procedure. The physical measurement in centimeters of the actual size of the CFs was done with further histological examination to verify the diagnosis. The following day, the hemostatic gauze was taken out from the vagina.

### 2.7 Additional literature review

Data on uterine cervical myomectomies were gathered through a comprehensive global English literature assessment, including case reports and original research publications (Supplementary Tables 3–9). Furthermore, study articles compiled from Chinese literature discussed cervical myomectomies (Supplementary Table 10). The search was limited to cervical fibroids, specifically myomas or leiomyomas. Hysterectomy and trachelectomy were the exclusion criteria. Cases involving vaginal enucleation of CFs, laparotomic or laparoscopic approaches, and subsequent hysterectomy, as well as conservative management of CFs or hysterectomy during Cesarean sections in obstetric patients, were also excluded. All other types of cervical neoplasms, including lipoleiomyoma, were also excluded. When various data, such as uterine body myomas or other surgical procedures, were included in the publications, only cases of cervical myomectomy were extracted. The size of CFs in centimeters was used for analysis. Finally, to avoid bias, only myomectomy cases were selected from all sources (case reports and cohort studies), and individual variables comparable to our findings from patients with CFs were used for statistical analysis grouped depending on surgical approaches, subsequently via vaginal, laparoscopic, and laparotomic ways of surgery in our comprehensive analyses. In addition, for this reason (to avoid bias), only analogous, comparable parameters from cohort studies were used. Subsequently, all sources were translated into English and presented in the reference list in the case report groups from English literature (Supplementary Tables 6–9) and in the cohort Chinese study groups (Supplementary Table 10). Parameters of 1,204 cases from the literature were available for comparative analysis. The analysis included row variables of 79 women from 78 case reports (Supplementary Tables 6–9) and 72 patients from five cohort studies: Wang et al. (32) (*n* = 12), Higuchi et al. (34) (*n* = 7), Matsuoka et al. (38), (*n* = 16), Sinha et al. (39), (*n* = 24), Zhang et al. (40), (*n* = 13) and summarized myomectomy outcomes of 117 patients from five cohort studies: Chang et al. (23); Kaneda et al. (25); Tian and Hu (27); Lee et al. (36); Takeuchi et al. (41). Data from 904 patients of the Chinese literature (Supplementary Table 10) with summarized mean and SD values on vaginal (Supplementary Table 10a: *n* = 282), laparoscopic (Supplementary Table 10b: *n* = 431), and laparotomic (Supplementary Table 10c: *n* = 191) myomectomies were compared with the current study outcomes (Supplementary Table 3). Variables of two studies were presented analogously in their original articles as the mean with range: Dou and Zhang (24) and median with 25–75% interquartile range: Hsiao et al. (35) (Supplementary Table 3).

### 2.8 Statistical analysis

Variables collected in the Excel table were transferred to the software (Graph Pad Prism. 10.4.1) format for statistical analysis. We identified outliers and cleaned data using the outlier identification test ROUT (Q = 1%) to ensure the accuracy of the data. Several outliers from measures of age, fibroid size, surgical time, and blood loss parameters were removed from the further statistics. The package of normality and lognormality tests (D’Agostino and Pearson test, Anderson-Darling test, Shapiro–Wilk test, Kolmogorov–Smirnov test) and the normal QQ plot of individual samples were used to identify the type of samples for further statistical analysis.

A Nonparametric Kruskal-Wallis test with Dunn’s multiple comparisons test was applied for samples grouped into three groups of the retrospective analysis of medical records of 32 patients because of the identification of the small sample size of the CCFL group (*n* = 7). A nonparametric Friedman’s and Dunn’s multiple comparisons test was applied to samples achieved by repeated measures.

An additional statistical analysis was performed aimed to compare current data (*n* = 32) with extracted individual variables from the worldwide English literature of 151 patients who had undergone cervical myomectomy published in 5 cohorts (32, 34, 38–40) studies (*n* = 72) and 78 case reports (*n* = 79) grouped in four groups and two subgroups. Statistical comparisons of extracted individual parameters were grouped depending on applied tests. A parametric Brown-Forsythe and Welch ANOVA test with Dunnett’s T3 multiple comparisons test for *n* < n50/group was used for samples that passed normality tests, a nonparametric Kruskal-Wallis test with Dunn’s multiple comparisons test for the samples that did not pass the normality tests and with small sample size or analogously unpaired two-tailed Mann Whitney test was applied for categorical variables. Available means and standard deviations (SDs) of five studies (23, 25, 27, 36, 41) and data from the Chinese literature were compared using parametric unpaired two-tailed *t*-test with Welch’s correction for samples with unequal SDs.

We performed post-hoc power analysis and sample size calculation for nulliparous incidence using an online post-hoc power calculator.^1^ We compared dichotomous endpoints of two independent studies (Nulliparous incidence, %), number of patients in groups at the alpha (0.05), beta (0.2), and power (0.8).

Results of individual parameters were presented in the Supplementary Tables as the mean and SD values and 95% upper/lower confidential intervals (CI) of the mean as well as minimum/maximum and range. *p*-values (<0.05) were considered significant with alpha = 0.05.

## 3 Results

### 3.1 Demographic factors

**Figure 3:**
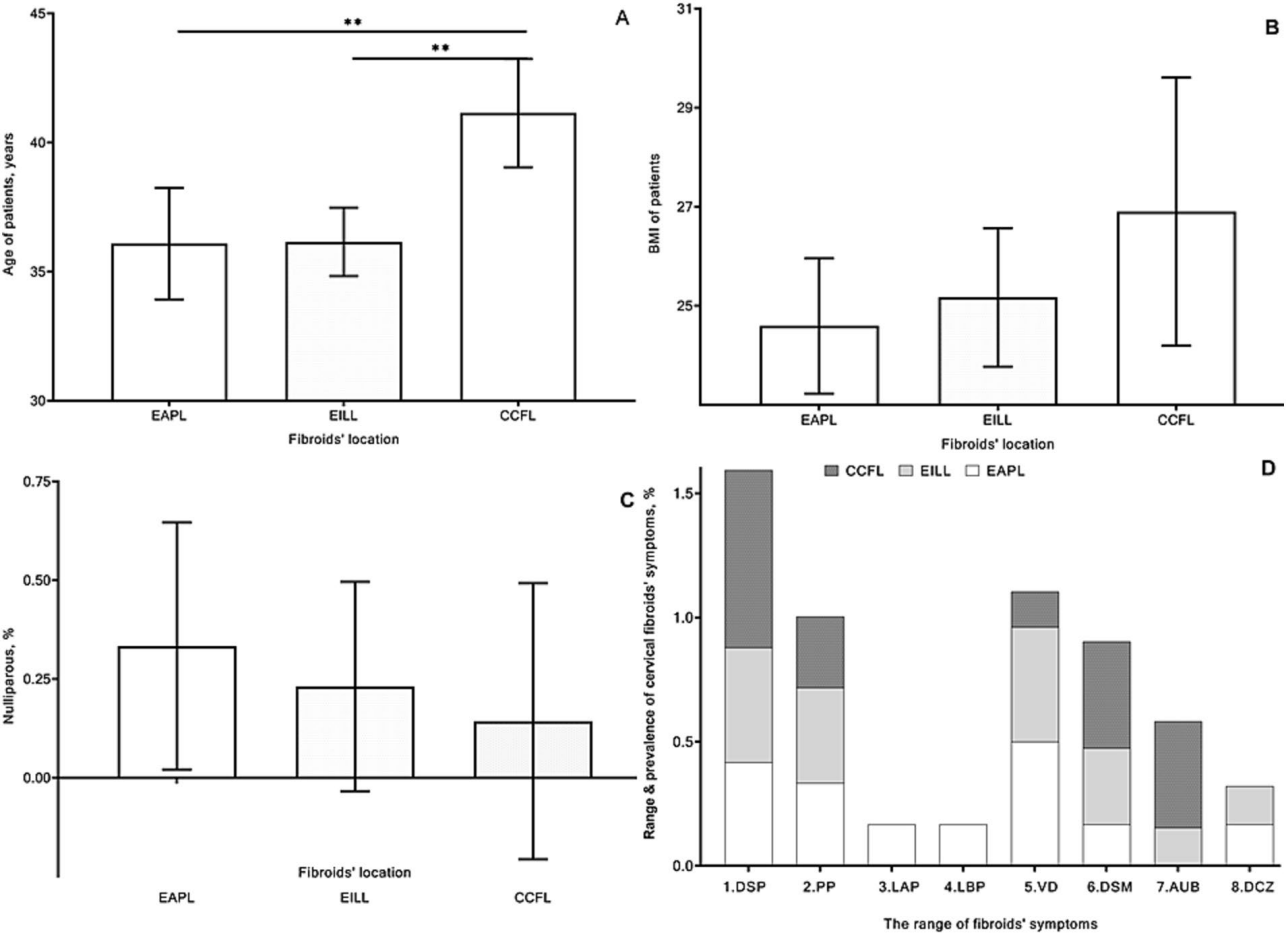
Mean age [**(A)** years], body mass index **(B)**, frequency of nulliparous women [**(C)** %], and range and prevalence of cervical fibroids’ symptoms [**(D)** %] of patients in group 1 with extracervical anterior–posterior localizations (EAPL), group 2 with extra- and intracervical lateral localizations (EILL), group 3 with combined cervical fibroid localizations (CCFL) of cervical fibroids. A nonparametric Kruskal-Wallis and Dunn’s multiple comparisons tests **(A–C)** and a nonparametric Friedman’s and Dunn’s multiple comparisons **(D)** tests were used and *p* values < 0.05 considered significant. 1. DSP, dyspareunia; 2. PP, pelvic pain; 3. LAP, lower abdominal pain; 4. LBP, lower back pain; 5. VD, vaginal discharge; 6. DSM, dysmenorrhea; 7. AUB, abnormal uterine bleeding; 8. DCZ, dyschezia.

The mean age was 36.08, 36.15, and 41.1 years, respectively, in groups 1, 2, and 3 with lower/upper 95% CI: 33.93/38.24; 34.83/37.48; 39.05/43.24 years (Figure 3A). The overall differences between groups by the Kruskal-Wallis test were significant (*p* = 0.003). According to Dunn’s multiple comparisons test, patients in the 1st and 2nd groups were significantly younger than those in the 3rd group (*p* = 0.009 and *p* < 0.01).

Parameters of BMI (Figures 3B) were not substantially different between groups. The mean and SD values of the nulliparous ratio were 0.33 ± 0.49, 0.23 ± 0.4, and 0.14 ± 0.38 without valuable differences between groups (Figure 3C).

### 3.2 Cervical fibroids’ clinical manifestation

Mainly the CFs were clinically manifested by a wide range of symptoms (Figure 3D), including dyspareunia, vaginal discharge, pelvic pain, and dysmenorrhea, respectively, in 16, 13, 11, and 9 cases. Abnormal uterine bleedings were found in 5 patients and four cases of lower abdominal and back pain, as well as four women who experienced dyschezia.

According to Friedman’s and Dunn’s multiple comparison tests, the sum and overall symptoms of the three groups were not significantly different (P=NS).

### 3.3 Cervical fibroids’ localization sites

We found 39 fibroids localized in the cervix, including two combined CFs in 7 patients. Single CFs were registered in EAPL in 12 (30.77%) of the total CFs’ count, arising from anterior and posterior lips of the cervix, respectively, in 7 (17.95%) and 5 (12.82%) cases in the 1st group. Single CFs in EILL were found in 13 (33.33%) women with extracervical and intracervical growth, respectively, in 8 (20.51%) and 5 (12.82%) cases in the 2nd group. 14 (35.9%) CFs were found among patients in 3rd group with CCFL:

- Extracervical anterior and intracervical posterior sites, *n* = 6 (15.38%);
- Right and left extracervical lateral sites, *n* = 4 (10.26%);
- Extracervical anterior and extracervical lateral sites, *n* = 2 (5.13%);
- Extracervical and intracervical posterior sites, *n* = 2 (5.13%).

In general, an extracervical direction was the most typical CF growth of CFs in 30 (76.92%) cases, whereas intracervical direction was only in 9 (23.08%) cases. 18 (46.15%) CFs were localized laterally with 13 (33.33%) extra and 5 (12.82%) intra-cervical growth, and among 17 (43.59%) extracervical CFs, 11 (28.21%) localized in the anterior and 6 (15.38%) in posterior lips of cervix. 4 (10.26%) intracervical CFs were attached to the posterior lips.

### 3.4 Cervical fibroid size (removed sample size)

**Figure 4:**
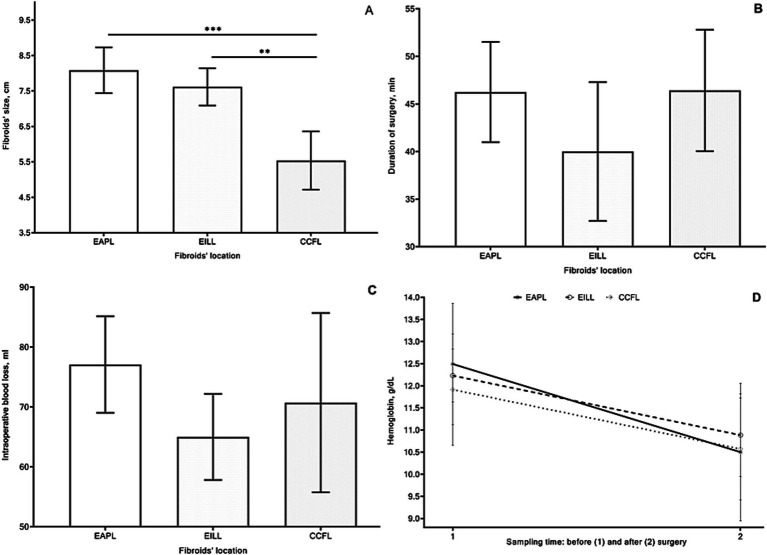
Cervical fibroid size [**(A)** cm], duration of surgery [**(B)** min], blood loss parameter [**(C)** mL], dynamics of hemoglobin levels before and after surgery **(D)** of patients in group 1 with extracervical anterior–posterior localizations (EAPL), group 2 with extra- and intracervical lateral localizations (EILL), group 3 with combined cervical fibroid localizations (CCFL) of cervical fibroids. Kruskal-Wallis and Dunn’s multiple comparisons tests **(A–C)** and a nonparametric Friedman’s and Dunn’s multiple comparisons **(D)** tests were used and *p* values < 0.05 considered significant.

The mean CF’s size was 8.08, 7.61, and 5.36 cm, respectively, in groups 1, 2, and 3 with lower/upper 95% CI: 7.44/8.73; 7.09/8.14; 4.71/6.35 cm (Figure 4A). There were overall significant differences between groups by the Kruskal-Wallis test (*p* < 0.001). The size of CFs in patients of the 1st and 2nd groups was significantly greater than that in the 3rd group (*p* < 0.001 and *p* = 0.007) by Dunn’s multiple comparisons tests.

### 3.5 Surgery time

The mean operation time, depending on the CFs localization site, was 46.25, 40.0, and 46.43 min, respectively, in groups 1, 2, and 3, with lower/upper 95% CI: 40.98/51.52; 32.7/47.3; 40.05/52.81 (Figure 4B) without substantial differences.

### 3.6 Intraoperative blood loss with repeated hemoglobin measurements

The mean of intraoperative blood loss was 77.08, 65, and 70.71 mL, respectively, in groups 1, 2, and 3 with lower/upper 95% CI: 69.02/85.15; 57.81/72.19; 55.75/85.68 mL (Figure 4C).

The blood loss parameter in patients of the 2nd group was less than that in the other two groups, without substantial differences. There were three cases of blood transfusion in two patients in the 1st group and one woman in the 3rd group. The mean values of hemoglobin were in EAPL (12.49/10.5), EILL (12.23/0.88) and CCFL (11.91/10.57) g/dL, respectively before/after surgery with lower/upper 95% CI: EAPL (11.62–13.36/9.51–11.49), EILL (11.87–12.59/10.32–11.45) and CCFL (10.75–13.08/9.51–11.64) (Figure 4D).

The Friedman test did not reveal a significant effect of the sum of Hb parameters (P=NS), and Dunn’s multiple comparisons test also did not demonstrate significant differences between groups (P=NS).

### 3.7 Hospital discharge day

Since patients tolerated the surgery well, three women from the 1st group and one from the 3rd group were discharged on the second day. In contrast, all other patients were discharged within 24 h after surgery, with a mean and SD of 1.12 ± 0.34 days for all patients.

### 3.8 Postsurgical follow-up

The long-term follow-up was > 3 years for sixteen patients, > 2 years for four patients, > 1 years for seven patients, < 1 year for five patients. Therefore, the long-term follow-up results were not summarized for all 32 patients.

**Figure 5:**
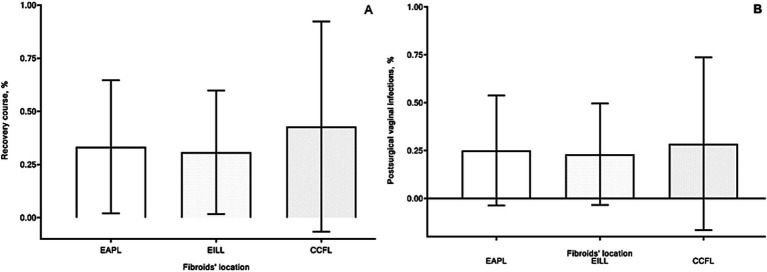
Recovery course [**(A)** %] and postsurgical minor local complications [**(B)** %] of patients in group 1 with extracervical anterior–posterior localizations (EAPL), group 2 with extra- and intracervical lateral localizations (EILL), group 3 with combined cervical fibroid localizations (CCFL) of cervical fibroids. A nonparametric Kruskal-Wallis and Dunn’s multiple comparisons tests were used and *p* values < 0.05 considered significant.

Resolution of CFs symptoms was observed in all cases. There were low hemoglobin levels observed in four patients from each 1st and 2nd groups and in three women from 3rd group because of intrasurgical blood loss and long-lasting symptoms of CFs (dysmenorrhea and abnormal uterine bleeding). However, there were no significant differences in hemoglobin levels before and after surgery between groups. Postsurgical recovery of all patients was uneventful, and mild postsurgical fever was observed without substantial differences between groups (Figure 5A). In several cases, local vaginal and cervical minor complications, such as postsurgical inflammatory reactions and bleeding, were observed: in 5, 4, and 2 cases, respectively, in 1, 2, and 3 groups.

Vaginal infections were observed in eight patients, three cases in each (1 and 2) groups and two cases in the 3rd group (Figure 5B). There were no substantial differences concerning these events between groups. All these complications were treated successfully in outpatient clinics.

All patients could enter their routine and start working ten days after hospital discharge. The long-term follow-up results generated by one-year clinical checks with TUS and annual telephone interviews (up to three years) showed two cases of extracervical posterior fibroids’ recurrences. Eight women became pregnant, and two patients decided to terminate pregnancies at 7–9 weeks of gestation for medical reasons. Five pregnancies were terminated full-term births, and one case was at 37 weeks of gestation without complications.

### 3.9 Comparisons of the current study findings with data from the literature

The secondary conclusions of the present study were drawn by comparing the current study results with analogous measures of 1,204 cases of cervical myomectomy from various populations worldwide. Data from the literature, including case report studies (VMEGP, VMEOP, LSME), LTME (LTGP/LTCS), and cohort studies (23–25, 32, 34–36, 38–41), including data from the Chinese literature were grouped into 21 samples formatted for statistical comparisons depending on type of surgical procedures such as VME, LSME and LTME, with grouping them to suitable parametric or nonparametric statistical tests.

#### 3.9.1 The population age

The current study population’s mean age was 37.22 ± 3.77 with a median of 37 years with the lower/upper limits of 31–44 years. CFs were identified in a variety of age groups in the reviewed literature, including two 17-year-old single girls among case reports: VMEGP and LTGP (Supplementary Tables 6, 9) and postmenopausal women in their 50s in several studies, including case reports VMEGP (Supplementary Table 6) and cohort studies (23, 24, 36). Most cohort studies’ mean and median age data fell within the range of women in the second half of their thirties (Supplementary Tables 3, 4).

The mean age difference between our retrospective sample and overall data from the English literature (7 comparisons), which passed normality tests, was very significant (*p* < 0.001) achieved using parametric Brown-Forsythe and Welch’s ANOVA tests (Supplementary Table 4). Significant and substantial age differences were found among case studies: VMEOP (*p* < 0.001); LTME (*p* < 0.001); subgroup LTGP (*p* = 0.018), and sample from the cohort study (32) (*p* = 0.002) compared to our retrospective sample by the Dunnett’s T3 multiple comparisons test (Supplementary Table 4). Analogously, there was a significant difference between the current study and samples from the literature (3 comparisons), in which data did not pass the normality tests (*p* = 0.026) using the nonparametric Kruskal-Wallis test. Among case reports, the women who experienced myomectomies during Cesarean sections (LTCS) were significantly younger compared to patients from the current study (*p* = 0.01) by using Dunn’s multiple comparisons test (Supplementary Table 4). Further, five English and three Chinese literature samples were analyzed using the two-tailed unpaired *t*-test with Welch’s correction. Among them, significant differences were found only for two cohort studies: Tian and Hu (27), (*p* = 0.013) and Chinese LSME (*p* = 0.019).

Comparisons of the age of patients of the current study to the population from the worldwide literature demonstrated that younger women underwent different types of myomectomy procedures among case reports (VMEOP, LSME, LTME, LTGP, LTCS) and in three cohort studies (27, 32) and ChLSME. There was only one cohort study by Hsiao et al. (35) with patients over 40. Approximately the same age population was observed among 11 compared samples, women in their late thirties, as the age of our patients (Supplementary Tables 3, 4).

#### 3.9.2 The parous status

The percentage of nulliparous women was another characteristic of the population. This parameter was obtained by counting nulliparous as one and parous as zero. The frequency of nulliparous women among our sample was 25% (Supplementary Table 3). At the same time, the frequency of nulliparous women ranged from 15.4 to 75.0% among nine samples available for statistical comparisons using an unpaired two-tailed Mann–Whitney test (Supplementary Table 4). There was a significantly high frequency of nulliparous women among case report studies: VMEOP (*p* = 0.048), LSME (*p* = 0.02), LTME (*p* = 0.013), LTGP (*p* = 0.004), and cohort study by Matsuoka et al. (38) (*p* < 0.001) compared to our data (Supplementary Table 4).

#### 3.9.3 Patients’ common complaints and queries

Our patients experienced a wide range of symptomatic CFs, so indications for surgery were related to these symptoms (Figure 3D). The population was mostly willing to preserve fertility with surgery for symptomatic CFs (Supplementary Table 5).

A wide range of symptoms were reported in the literature, depending on the size and growth direction of the CFs. Depending on the size of the fibroids, there were various symptoms of chronic pain anxiety, including dyspareunia, pelvic pain, stomach pain, lower abdominal pain, severe pain during sexual activity, deep dyspareunia producing extreme misery impeding everyday tasks, and persistent cramping similar to menstruation. Menorrhagia, irregular menses, hypermenorrhea, heavy menstrual bleeding, vaginal discharge, bleeding and vaginal discharge, severe heavy menstrual bleeding, menometrorragia, heavy menstrual and intermenstrual bleeding, dysmenorrhea, and vaginal discharge with smelling are some of the symptoms that have been linked to bleeding and menstrual disorders (Supplementary Table 5).

The growth of CFs brought on abdominal fullness and pressure symptoms, such as swelling and heaviness in the lower abdomen, palpable mass in the belly and pelvis, expanding lump in the abdomen, increasing abdominal girth or lump (mass), and full pelvic pressure. The growth direction of CFs was found to affect the physiological habits of the bladder and colon, including frequent urination, intermittent urine retention and urgency, tenesmus, constipation, dyschezia, urinary incontinence, urgency, and frequency.

Severe CFs patients who were not treated were reported to have symptoms such as a protruding mass into the vagina, a mass extrusion in the vaginal opening with foul-smelling discharge, a protruding mass out of the vagina, and a hanging mass that made walking difficult. Large CFs resulted in severe pregnancy and delivery difficulties, including miscarriage, early pregnancy vaginal discharge and bleeding or late pregnancy and delivery hemorrhage, and postpartum issues. Lastly, CFs can cause anemia, chronic fatigue, and nonspecific symptoms that can lead to infertility (Supplementary Table 5).

#### 3.9.4 Fibroid size

Our patients’ CF sizes ranged from 7.01 ± 1.59 cm to 7.5 (4–9) cm, with a median and lower/upper limit (Supplementary Table 3). In younger individuals, we discovered a correlation between larger CFs and solitary growth as opposed to two merged fibroids. Among case report studies involving vaginal myomectomies, the size of CFs with extreme deviations of the rank limitations (ranging from 2.5 to 38 cm) was found (Supplementary Tables 6, 7). Compared to the current study parameter, most cohort studies showed similar values. They did not differ substantially (Supplementary Table 4). Although Brown-Forsythe (*p* = 0.033) and Welch (*p* < 0.001) ANOVA tests revealed significant differences between our data compared to seven samples from the literature only one cohort study sample (32) was demonstrated individual significance by the Dunnett’s T3 multiple comparisons test for *n* < n50/group (*p* < 0.001). Analogously high significant differences of six samples compared to our data (Supplementary Table 4) achieved by the Kruskal-Wallis test (*p* < 0.001) were confirmed with significantly bigger sizes of CFs only in two case report studies: LTME and LTGP (*p* < 0.001). Among six studies compared unpaired two-tailed *t*-tests with Welch’s correction for samples with unequal standard deviations, significant differences were observed only for two Chinese samples, ChVME (*p* < 0.001) and ChLT (*p* = 0.022).

#### 3.9.5 The surgery time

This investigation demonstrated that the benefit of our transvaginal CIME technique was a shorter surgical time. The average surgical duration was 43.75 ± 10.0 min, with a 45-min median and lower- and upper-bound ranges of 20–60 min (Supplementary Table 3).

All comparisons of the Brown-Forsythe and Welch ANOVA with Dunnett’s T3 tests showed significant differences (*p* < 0.001) between our data and five cohort study samples: Sinha et al. (39) with/without UAL (32, 40). The summary of five studies using the Kruskal-Wallis test and three samples among them, LSME and cohort studies (34, 38) by using Dunn’s multiple comparisons test showed significant differences compared to our data (*p* < 0.001). Individual comparisons by using an unpaired two-tailed *t*-test with Welch’s correction showed substantial differences for the cohort study (23), all Chinese samples (*p* < 0.001), and for the study by Lee et al. (36) (*p* < 0.007) (Supplementary Tables 3, 4).

The current study’s surgical time characteristics were substantially shorter than those of all the examined studies from the literate population (Supplementary Table 3). In comparison to the current study parameter, all evaluated samples LSME (23, 32, 34, 36, 38–40), and Chinese projects had longer surgical times. In other research (24, 35, 41), the median values with lower upper ranges were also substantially longer (Supplementary Table 3).

#### 3.9.6 Intraoperative bleeding

For surgeons who are willing to do vaginal myomectomy, the primary unresolvable problem is intraoperative bleeding in cases where the surgical field is small and encircled by vital anatomical systems like the bladder, rectum, and ureters. In the current study, the blood loss was 70.78 ± 13.86 mL on average, with a median of 75 mL and lower-upper limits of 50–95 mL (Supplementary Table 3). Comparisons by using the Brown-Forsythe and Welch ANOVA with Dunnett’s T3 tests showed significant differences (*p* < 0.001) between our data and five cohort study samples (Supplementary Table 4). Among them, individual significant differences were for two cohort studies: (*p* < 0.001) (32) and (*p* = 0.002) (40). The summary of eight studies using the Kruskal-Wallis test demonstrated significant differences compared to our data (*p* < 0.001) with analogous changes in two cease report studies: LTME (*p* < 0.001); LTCS (*p* = 0.019). Analysis of seven individual samples by using an unpaired two-tailed *t*-test with Welch’s correction revealed significant differences (*p* < 0.001) of six cohort studies compared to our data, including four cohort studies (23, 27, 36, 41); and all Chinese studies (Supplementary Table 4).

Numerous studies’ blood loss values were significantly higher than the amount lost in this study. Nonetheless, there was a substantial rise in blood loss values in the cohort study LSME and among Cesarean studies (Supplementary Table 4). In three studies, reduced blood loss was more substantial than that of the current research (Supplementary Tables 3, 4): application of LLT and VPI in 12 patients (32), only VPI in 13 patients (40), and a combination of the uterine artery clamping and VPI in 5 LTMEs (41).

#### 3.9.7 Perioperative blood loss prevention management

There was a miscellaneous character of perioperative blood loss prevention management (Supplementary Table 5). Cohort studies planned to treat CFs prescribed to their patients Gonadotropin-releasing hormone agonists up to 6 months before surgery (23, 34, 36, 38, 41, 42). A diluted vasopressin injection is the primarily used blood loss prevention management worldwide: case report study Supplementary Tables 5–8; cohort studies (23, 32, 34–36, 38, 40, 41). Other medications, such as terlipressin (24) and epinephrine, were also applied in Chinese studies (Supplementary Table 10). Oxytocin injection during Cesarean sections was also aimed to prevent bleeding during and after surgery: LTCS (27). In studies designed in hospitals with advanced endovascular technologies, an embolization or temporary balloon occlusion of the uterine and internal iliac arteries and other sophisticated methods were also applied (43). Surgeons, depending on their skills, performed temporary bilateral internal iliac artery or uterine artery balloon occlusion, uterine artery clipping, or ligation: LTME (23, 39, 41). Some surgeons used the ‘water caution method’ to reduce blood loss during surgery (24) or a local hemostatic barrier to fill the fibroid bed to prevent bleeding after surgery: LSME (44). Finally, the individual surgical skills of surgeons and their experience with such cases, if they had appropriately equipped surgical rooms, play an essential role in blood loss. Sinha et al. (39) showed reduced blood loss in patients with uterine artery ligation before myomectomy compared to those without ligation in a matched cohort study. There was a significant difference between the current study and the group without uterine artery ligation (*p* = 0.0021), showing increased blood loss among these patients (Supplementary Table 3). In contrast, there were no substantial differences between the uterine artery ligation group (39) and the current study results.

#### 3.9.8 The prolongation of hospital stay days

The mean of hospital stay days (HSD) of our patients was 1.12 ± 0.34 days with a median of 1 day and lower-upper limits of 1–2 days (Supplementary Table 3). Four cases with two HSDs identified as outliers among our patients were ignored. The most extended hospitalization of 15 days in an unmarried 22 years-old girl who experienced laparotomic myomectomy among case reports (Supplementary Table 9) was related to the excision of the sizeable CF of 30×25 cm arising from the anterior lip of the cervix and antibiotic treatment because of wound soakage on the fifth postoperative day. Another case of the most prolonged hospitalization of 20 days was among 16 patients with CFs who suffered the Cesarean section (LTCF) (27). It was unclear which puerperal woman experienced 4,200 mL hemorrhagic blood loss when myomectomy was in nine patients and hysterectomy in three cases among these 16 patients. Therefore, this study’s HSD and blood loss outcomes were excluded from the analysis (27). There were highly significant differences (*p* < 0.001) in the HSD of six case report samples: VMEGP, VMEOP, LTME, LTGP, LTCS, cohort study (32) and LSME (*p* < 0.001) compared to or data by using the Mann Whitney test (Supplementary Table 4).

The HSD of all other studies was substantially longer than that of the current research (Supplementary Tables 6–9) and two cohort studies (23, 32). Among individual Chinese studies, the shortest and longest mean HSD was 4.06 ± 1.22 and 11.3 ± 1.6, with more extended hospitalization in all three ways of myomectomy than in the current study (Supplementary Tables 3, 4).

## 4 Discussion

The primary conclusions were based on findings from the retrospective study of 32 patients.

The general conclusions of this comprehensive investigation were drawn from comparing the retrospective findings with the broad literature data, which included 1,204 patients worldwide.

### 4.1 The primary conclusions of a retrospective study

This retrospective study unveils a novel surgical procedure, vaginal myomectomy, which was successfully performed on a series of 32 patients (Supplementary Table 2). These comparisons were based on fibroid localization and size, as well as single or combined cases of CFs in three groups of patients. The aim was to evaluate the feasibility of conducting CIME via the transvaginal approach.

While analyzing demographic factors, we detected larger-sized CFs (*p* < 0.001) in younger patients (*p* = 0.003) among individuals presenting single fibroids compared with women boring combined two CFs. All other presurgical measurements did not show any conclusive differences between groups.

Thirty extracervical fibroids (30/39) were the most frequent and typical localization of CFs, whereas intracervical fibroids were found less often, only in nine cases among 39 CFs. Notably, the localization of CFs in anterior/posterior lips and lateral cervical sites was equal, with 18 and 17 cases, respectively. We found twenty-five single and fourteen combined CFs. Finally, based on these observations, an original classification of CFs was developed.

There were no substantial differences between groups concerning peri-surgical (time, blood loss, hospital discharge day) and postsurgical follow-up parameters. The outcomes showed that vaginal myomectomy of CFs may be done regardless of the size and location of CFs subsequently, all three tested hypotheses were rejected.

### 4.2 The frequency of the cervical fibroids

The worldwide generally accepted opinion that CFs are a rare condition is based on previous histological findings by Tiltman (21), who found only four (0.6%) CF samples among 661 hysterectomy specimens in postmenopausal women in the South African Institute for Medical Research. However, a close analysis of the literature may shed light on this underestimated aspect of the problem (Table 1). Further, in two studies for premenopausal women, de Bruijn et al. (13) from the Elisabeth Tweesteden Hospital (Tilburg, Netherlands) and Kim et al. (45) from the Research Institute of Radiological Science (Seoul, Korea) performing uterine artery embolization observed twelve (1.02%) and ten (1.86%) cases of CFs, respectively, among 1,180 and 537 women with symptomatic uterine myoma or adenomyosis (13, 45). Meanwhile, the ranged frequency of CFs from 3.74 to 4.9% was observed in four studies with myomectomies and hysterectomyies: In the Juntendo University Hospital (Tokyo, Japan), Kaneda et al. (25) identified twenty two large CFs (3.74%) filled in the pelvic cavity among 823 patients with uterine myoma and performed ten abdominal myomectomies and twelve total abdominal hysterectomies with internal iliac artery balloon occlusion; Ingala et al. (26) observed 26 (4.04%) individuals with CFs among 644 Congolese women receiving treatment in three medical facilities for myomectomy and hysterectomy in the capital city (Brazzaville, Congo); Taniguchi (46) while performing total laparoscopic hysterectomy in pre and postmenopausal women diagnosed 21 (4.5%) large symptomatic CFs among 463 women in the Takanohara Central Hospital, (Ukyo Nara City, Japan); Takeda et al. (47) found thirteen cases (4.9%) of large symptomatic CFs among 267 uterine myomas and performed temporary endovascular balloon occlusion of the bilateral internal iliac arteries to control hemorrhage during laparoscopic-assisted vaginal hysterectomy in the Prefectural Tajimi Hospital (Gifu, Japan).

**Table 1:** Frequency of cervical fibroids (CFs) among surgically treated (myomectomy/hysterectomy) patients with uterine myoma and women who have undergone uterine artery embolization to eliminate myoma or reduce its size.

Authors	Facilities	Study subject	Population (mean/median with min/max years)	Total, *n*	Frequency of CFs, *n* (%)
1. Tiltman (21)	South African Institute for Medical Research	Hysterectomy specimens	Gynecological patients with hysterectomy: 41 (12–84)	661	4 (0.6)
2. de Bruijn et al. (13)	Elisabeth Tweesteden ziekenhuis, Tilburg, Netherlands	Uterine Artery Embolization	Women in their late 30s: 37.5 (33–47) with symptomatic myoma	1,180	12 (1.02)
3. Kim et al. (45)	Research Institute of Radiological Science, Seoul, Republic of Korea	Uterine Artery Embolization	Women in their 40s: 40.5 (28–50) with symptomatic fibroids or adenomyosis	537	10 (1.86)
4. Kaneda et al. (25)	Juntendo University Hospital in Tokyo, Japan	LT myomectomy/hysterectomy with IIABO	Women in their late 30s: 38 (22–52) with large CFs filled the pelvic cavity	823	22 (3.74)
5. Ingala et al. (26)	Three capital city medical facilities, Kinshasa, Congo	Myomectomy and hysterectomy	Women in their late 30s: 38 (24–45) with myomas	644	26 (4.04)
6. Taniguchi (46)	Takanohara Central Hospital, Ukyo, Nara City, Japan,	Total laparoscopic hysterectomy	Pre/post-menopausal women: 46 (37–65) with large symptomatic CFs	463	21 (4.5)
7. Takeda et al. (47)	Gifu Prefectural Tajimi Hospital, Japan	LS-assisted vaginal hysterectomy with IIABO	Pre/post-menopausal women: 45 (36–56) with large symptomatic CFs	267	13 (4.9)
8. Bajo et al. (22)	Getafe University Hospital in Madrid, Spain	Histologically confirmed CFs	Postmenopausal women: 50 (47–60) with benign cervical conditions	290	21 (7.24)
9. Dou and Zhang (24)	Medical University Hospital in Shenyang, China	LS & robot-assisted myomectomy	Women, their late their 30s: 36.5 (23–52) with symptomatic CFs	235	18 (7.66)
10. Chang et al. (23)	National Taiwan University Hospital, Taiwan	LS myomectomy with uterine artery ligation	Women in their late 30s: 38 (24–52) with large symptomatic CFs	367	28 (7.68)
11. Hsiao et al. (35)	Far Eastern Memorial Hospital, New Taipei City, Taiwan	Robot-assisted & LS myomectomy	Pre/post-menopausal women: 47.5 (43–48) with symptomatic myoma	42	14 (33.3)

CFs, Cervical fibroids; IIABO, internal iliac artery balloon occlusion.

Another three studies demonstrated comparably higher cases of CFs (7.24–7.68%): Bajo et al. (22) reported histologically confirmed CFs in 21 (7.24%) samples out of 290 specimens in the Getafe University Hospital (Madrid, Spain); Dou and Zhang (24) identified 18 (7.66%) cases of CFs among 235 patients who had laparoscopic and robot-assisted myomectomy at the Medical university hospital (Shenyang, China); Chang et al. (23) reported 28 (7.68%) CFs among 367 patients who had laparoscopic myomectomies at the National Taiwan University Hospital.

The extremely high cases of symptomatic CFs in fourteen patients (33.3%) among 42 pre/postmenopausal women observed by Hsiao et al. (35) probably related to the impact of the specialized clinic with experienced surgeons in the Far Eastern Memorial Hospital (New Taipei City, Taiwan).

Among many published case reports describing CFs from India, a recently published retrospective cohort study that included 24 women drew our attention. Among them, VME was performed in 12 cases, LSME in 4 women, and laparotomic hysterectomy in 8 patients (48). Intriguingly, all these cases were observed in two years (from January 2021 to January 2023) at the obstetrics and gynecology department of the PGIMS, University of Health Sciences, Rohtak (Haryana, India). Unfortunately, we could not include the results of this study in our analysis because of the absence of similar variables for comparisons, and myomectomy cases were mixed with the hysterectomy (48). Publications in the global literature concerning CFs have mainly described particularly extraordinary severe cases and large fibroids with suitable illustrations for submission from hospitals equipped with modern diagnostic visualization tools. Doctors in these facilities are usually highly qualified and can solve patients’ problems with such complicated conditions. Therefore, most CF cases with usual conditions, in which doctors did not use appropriate diagnostic methods to get illustrations for publications, have remained unknown. The increasing number of studies on this subject due to the implication of modern visualization tools has shown that the prevalence of CFs is underestimated, taking into account the high prevalence of uterine myomas in overaged patients and among half of the reproductive-aged women (49, 50). This highlights the need for better diagnostic practices in all healthcare settings, not just in specialized hospitals with advanced tools.

### 4.3 Classification, clinical manifestations, and surgical management of cervical fibroids

#### 4.3.1 The origin and growth directions of the CFs with the depth of the lesions

Further, we analyzed the CF’s characteristics, such as their attached sites and growth directions, with the depth of the lesions in parallel with the patient’s complaints (Supplementary Table 5). We found that the anterior and posterior lips were the initial source of the majority of CFs, followed by the lateral borders of the cervix. They grew in extracervical, intracervical, or mixed directions. We did not classify the CFs in the current investigation as subserosal, submucosal, or intramural lesions because they were inside the cervical region. Comparably, the majority of studies, including case report studies (Supplementary Tables 6–9) [(23, 34, 36, 39, 40) and all Chinese studies] - classified CFs as anterior, posterior, and lateral based on their localizations and subsequent origin from anterior and posterior lips as well as left or right lateral edges of the cervix (Supplementary Table 10).

There were studies indicating the intracervical (Supplementary Table 3) or central location of CFs (23, 32), including the case report studies (Supplementary Tables 6, 7) or describing them as cervical myoma with their origin as the whole cervix (24, 25, 27, 35, 38, 41). Several studies reported the growth of CFs to the extracervical, intracervical, or combined directions (38), including case report studies (Supplementary Table 9) analogously to our description. Other studies (23, 25, 27) described an extended growth of CFs into the pelvic cavity, the broad ligament, the retro cervical or cervical region of the lower uterine segment, and the mass protruded into the vagina, including most case reports (Supplementary Tables 6–9). There is a wide range of growth depth descriptions of CFs as the mass within the cervix (23, 41), subserosal, submucosal, and intramural lesions, as well as their intramyometrium extension in cohort studies (23, 32, 34, 36, 38) and all Chinese literature (Supplementary Table 10). The lesion’s depth in many studies was combined with the growth directions when the description gives the view of the whole manifestation of CFs (Supplementary Table 5), for example, the mass filling the pelvic cavity (25, 35) and in case reports (Supplementary Table 9), deep-rooted cervical myoma with lesion within the cervix protruding into the vagina (23), the mass filling the vagina and the mass protruding from the vagina prolapsed and hanging in the case report studies (Supplementary Tables 6, 7).

#### 4.3.2 Classification of cervical fibroids

**Figure 6:**
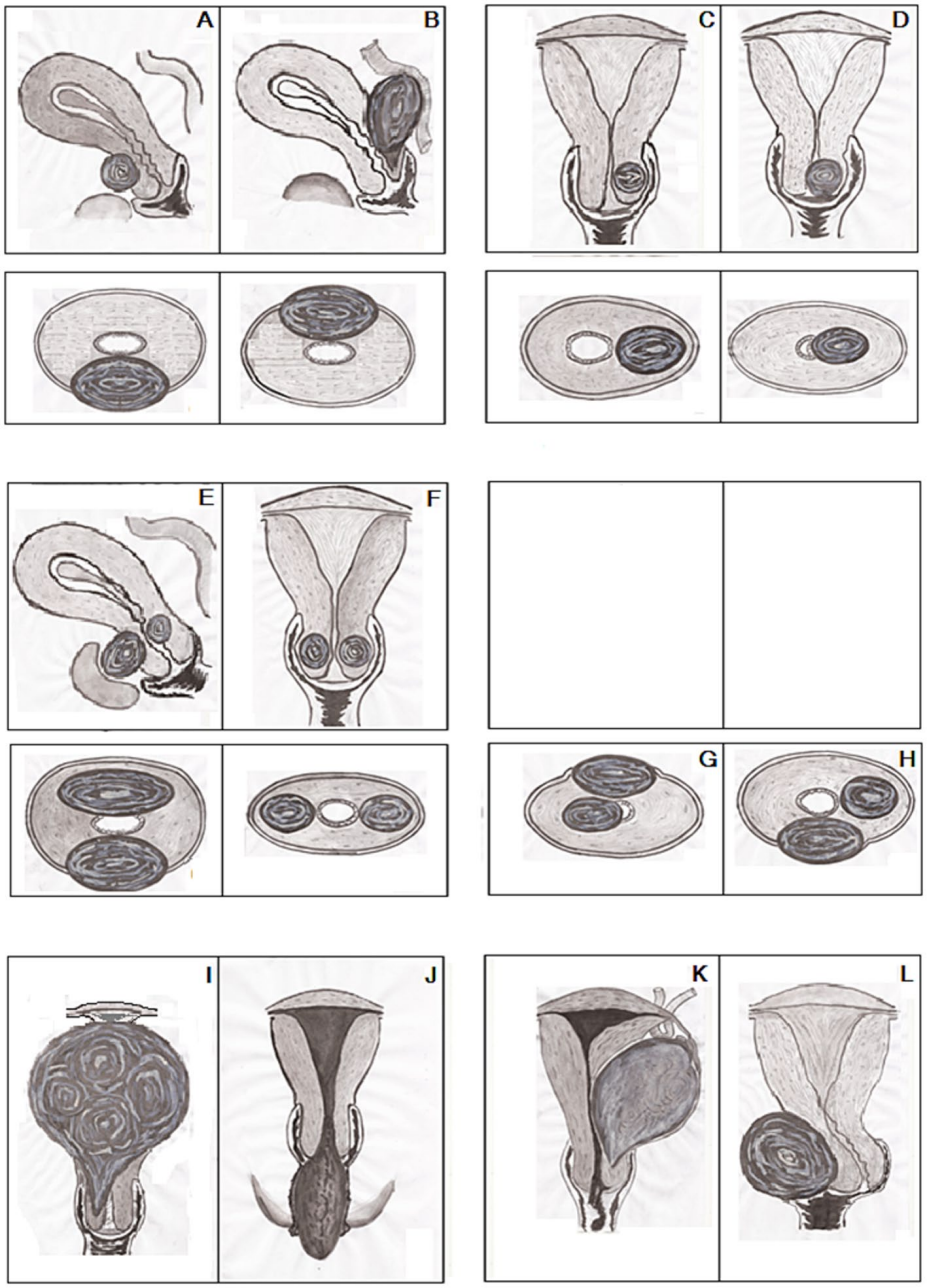
Classification of growing and mature cervical fibroids (CFs): **(A,B)** Extracervical anterior/posterior localizations (EAPL). **(C,D)** Extra/intracervical lateral localizations (EILL). **(E–H)** Combined cervical fibroid localizations (CCFL). **(I)** Central upward. **(J)** Central downward. **(K)** Lateral-upward. **(L)** Lateral-side growth of CFs. Upper part **(A–F)**—sagittal/frontal, lower part **(A–H)**—axial and **(G–L)**—frontal dimensions of the uterus with CFs.

Our descriptions of CFs entirely corresponded with the three types (A-C) of CFs according to the classification by Torpin and Beard (9). Subsequently, type A, fibroids originating from the upper cervical portion with the elevation of the uterine fundus above; type B, interstitially growing fibroids; type C, pedunculated fibroids within or protruding from the vagina and our interest was out of type D in the classification Torpin and Beard (9) since the current study did not include the fibroids arising from a remnant of the cervix. Accordingly, the findings of the current research and the analysis of 79 individual cases from 78 case report articles (Supplementary Table 3) and 72 women from five cohort studies (32, 34, 38–40) and the summarized data of 149 patients from other seven sources (23–25, 27, 35, 36, 41) (Supplementary Tables 6–9) we identified the CFs as ‘the growing fibroids’ when their size is not initiated severe acute life-threatening conditions or not disrupted basic daily routines of patients (Figure 6). We observed three types of growing CFs: Extracervical growth of CFs arising from anterior and posterior lips of the cervix, defined as extracervical anterior–posterior localizations (EAPL, Figures 6A,B); CFs originating from the lateral sides of the cervix with extracervical and intracervical growth are named extra and intracervical lateral localizations (EILL, Figures 6C,D). Combined cervical fibroid localizations (CCFL, Figures 6E–H) were observed in two positions in each case. We found their four combinations: (1). Extracervical anterior and intracervical posterior; (2). Extracervical right/left lateral positions; (3). Extracervical anterior and extracervical lateral locations; (4). Extracervical and intracervical posterior sites. Depending on their kinds and growth orientations, large-sized CFs may present with severe acute illnesses that need immediate surgical intervention to preserve the patients’ lives or allow them to resume their regular lives. Since these CFs have grown to maximum size, they produced changes and injured surrounding tissues and organs in the pelvic cavity, disabling women from their daily routines; we named these CFs “the mature fibroids” (Figures 6I–L).

Based on comprehensive literature analysis, we identified four types of these mature CFs affecting patients’ daily routines: CFs with central upward growth with filling the pelvic cavity and pulling up the body of the uterus (Figure 6I); CFs with downward growth with protruding into the vagina and prolapsing out of the vaginal orifice (Figure 6J); CFs with lateral upward growth initiating ovarian tumor clinical manifestations (Figure 6K); CFs with lateral side growth with pushing the cervix to the contrary lateral direction and filling the lower part of the pelvic cavity (Figure 6L). Subsequently, the principles of these classifications (9, 10) could be suitable for mature fibroids when the CFs reach their optimal size and typical locations with distinctive manifestations.

This CF classification is also in line with the division of CFs into anterior, posterior, lateral, central, and numerous categories by Monaghan et al. (10). Because of their typical look, the central growth of CFs was literary compared to a “Lantern on St. Paul’s dome” (10). The pedunculated CFs that protrude into and prolapse out of the vagina were not described, though. The proposed classification provides detailed features of growing fibroids with generalized symptoms and non-specific presentations and mature fibroids with unique symptoms and appearance.

A growing body of research showed that new visualization and therapy technologies enhanced physicians’ ability to manage patients with cervical fibroids. Regardless of the magnitude of the developing CFs, the primary indications for surgical therapy were symptomatic CFs and fertility preservation in non-symptomatic women. In light of this, our classification system is more valuable in the current context for facilitating an easier understanding of the underlying causes of CFs’ clinical manifestations based on the size, growth direction, and depth of lesions and for managing subsequent patient therapy.

#### 4.3.3 Clinical manifestations of cervical fibroids

Clinical manifestations of CFs depend on their locations and size. Consequently, pelvic/abdominal pressure symptoms, such as a growing belly lump, an increasing belly girth, abdominal swelling, heaviness in the lower abdomen, and pelvic/lower abdominal pain due to the increased size of CFs with the elevation of the uterine body above, were the typical complaints of patients with CFs type A by Torpin and Beard (9) and central by Monaghan et al. (10), according to our analysis (Supplementary Table 5). The interstitial growth direction of the mass, as described by Torpin and Beard (9) type B, affects the symptoms of cervical fibroids as well. The cervix’s anterior and posterior lips or lateral sites may be affected (10). An anterior extension of CFs was linked, in our analysis of case reports, to pressure on the bladder up to anatomical changes in the urinary system that were manifested as complaints of urgency, incontinence, difficulty micturition, frequent urination, or intermittent urinary retention (16–18).

Analogously, the increased size of posterior CFs with the pressure on the rectum produced symptoms such as tenesmus and constipation in case report studies (Supplementary Tables 6–9), whereas an extension to the lateral sites could imitate the symptoms of ovarian tumors (15, 18–20).

Walking difficulties and the typical complaints of something coming out or hanging out of the vagina were the manifestations of the pedunculated fibroids within or protruding from the vagina type C by Torpin and Beard (9). According to case reports, irritations, trauma, and inflammation of these prolapsed masses were manifested with bleeding and smelling vaginal discharge (Supplementary Tables 6–9). Analysis of case reports showed that the enormously increased size of CFs with their severe clinical manifestations required urgent surgical treatment through vaginal or open access depending on the type of CFs in case reports (Supplementary Tables 6–9). In contrast, manifestations of the growing CFs (Supplementary Table 5) widely ranged with such symptoms as vaginal discharge, abnormal vaginal bleeding, pelvic/abdominal pain, menstrual disorders, menorrhagia, dysmenorrhea, anemia due to hypermenorrhea, dyspareunia, bleeding after coitus, infertility, and miscarriages in case reports (Supplementary Tables 6–9) (23, 24, 34, 36, 38–40).

Comparisons of parameters of our technique with that of the laparoscopic LLT showed that Wang et al. (32) using a self-made Roeder knot technique and VPI in younger patients (*p* < 0.001), with bigger size of CFs (*p* = 0.03) willing fertility preservation demonstrated significantly decreased blood loss (*p* < 0.001). However, the other two perioperative parameters, mainly the surgery time and postsurgical hospitalization of patients, were significantly reduced (*p* < 0.001) in our patients compared with that of the study by Wang et al. (32).

Taking into account possible side effects and complications of myomectomies and hysterectomies in large series of observations (51, 52) and regarding perioperative outcomes, such as laparoscopic surgery under general anesthesia, endotracheal intubation with lung ventilation, and CO2 pneumoperitoneum, which triggers particular side effects and complications (53). These findings demonstrated that surgeons and patients should prefer vaginal CIME to treat CFs.

#### 4.3.4 Surgical approaches for cervical myomectomy

The cervical myomectomies were performed via LS, LT, and vaginal approaches (Supplementary Table 3), respectively, in 212 (70.66%), 44 (14.67%), and 44 (14.67%) cases among 300 patients in English literature (23–25, 27, 32, 34–36, 38–41), including case report studies represented in (Supplementary Tables 6–9). Analogously myomectomies were performed via LS, LT, and vaginal approaches, respectively, in 431 (47.68%), 191 (21.13%), and 282 (31.19%) cases in Chinese cohort studies (Supplementary Table 10). The LSME was the most applied technique of CFs treatment, especially among young populations independently of the study origin. One case of the LS approach was converted to open surgery (34). This patient was excluded from the LS group (34) and included in the case report study (Supplementary Table 9a). Analogously, two patients who experienced vaginal and laparotomic cervical myomectomies from the case series article were allocated to case report studies (Supplementary Tables 6–9). There were only 42 case reports describing 44 cases of vaginal myomectomies to treat CFs in the English literature between 1885 and 2022 (Supplementary Tables 6, 7). In contrast, ten Chinese cohort studies with 282 cases were published between 2007 and 2020 (Supplementary Table 10a). It should be noted that vaginal myomectomies in 29 gynecologic patients were performed because of symptomatic CFs in the case report study (Supplementary Table 6), whereas 15 in obstetric patients, to treat complications of pregnancy, delivery, and postpartum periods in the case report study (Supplementary Table 7). Analogously there was a high activity of Chinese researchers in a short time concerning LS and LT surgeries to treat CFs (Supplementary Tables 3, 10b,c). Several studies applied the robot-assisted LS technique to excision CFs (24, 31, 35). In the case report study (Supplementary Table 9), among 25 LTMEs, 21 cases were gynecologic patients (Supplementary Table 9a). Four myomectomies were simultaneous cervical myomectomies during Cesarean sections (Supplementary Table 9b). Another ten LTMEs come from a cohort study (25), and 9 cases were myomectomies performed during or after Cesarean sections (27). Finally, 44 cases of LTME were collected from the English literature, including cohort, case series, and case report studies.

### 4.4 Strengths

This retrospective study unveils a novel surgical procedure, transvaginal CIME, which was successfully performed on a series of 32 patients. The outcomes showed that transvaginal CIME of CFs may be done regardless of the location and size of CFs. A comparative analysis of the findings with perioperative endpoints of 643 laparoscopic, 235 open, and 326 vaginal myomectomies from the global literature demonstrated the viability and efficacy of the transvaginal CIME. Additionally, a comprehensive description of the various clinical manifestations of growing and mature CFs based on factors such as size, growth direction, localization sites, and depth of lesions was proposed, along with a classification of these patients. Depending on the size, growth direction, and depth of lesions, the classification may make it simple to determine the origin of CFs’ clinical manifestations and adjust patient care accordingly. Since the daily routines and reproductive objectives of patients are impeded by CFs, this novel transvaginal CIME may enhance patients’ well-being.

### 4.5 Limitations

The limitations of this study are evident in the low number of patients, a direct result of the low prevalence of CFs, as indicated by the scarcity of case reports in English literature. The multiplicity of analyses, while a strength, also presents a limitation because many observations were drawn from Chinese language literature, which may limit our readers’ access to the part of sources.

Additionally, we performed post-hoc power analysis and sample size calculation (Supplementary Table 11) for nulliparous incidence using an online post-hoc power calculator (54). We applied dichotomous endpoints of two independent studies (nulliparous incidence, %), number of patients in groups at the alpha (0.05), beta (0.2), and power (0.8) for two case report studies: LSME (*n* = 10), LTME (*n* = 21) and two cohort studies: Matsuoka et al. (38), (*n* = 16), Chang et al. (23), (*n* = 28) comparing to our data. Subsequently, post-hoc power was 80 or higher in three studies with sufficient actual sample size (Supplementary Table 11) to reject or confirm the study hypothesis (55), supporting our comparisons using literature data.

### 4.6 General summary

According to the findings, CFs with a mean size of 8.08 cm with lower/upper 95% CI (7.44–8.73 cm) in extracervical anterior and posterior sites, 7.61 cm (7.09–8.14 cm) in extracervical and intracervical lateral localizations, and 5.36 cm (4.71–6.35 cm) for combined CFs in two different localization sites could potentially be successfully treated with the transvaginal CIME. Through a comparative analysis of perioperative endpoints from 326 vaginal, 643 laparoscopic, and 235 open (laparotomic) myomectomies from the global literature, were demonstrated the feasibility and efficacy of the original CIME technique. Additionally, a unique classification of growing and mature CFs was proposed, along with detailed descriptions of their various clinical manifestations based on size, growth directions, localization sites, and depth of lesions. This classification could make it easy to understand the cause of the clinical manifestation of CFs depending on their locations, growth directions, size, and depth of lesions, with subsequent patient management accordingly. Our study results could increase the attention of practitioners to treat CFs through vaginal access by using current visualization diagnostic methods and also our classification of fibroid localization to manage patients with such severe medical conditions in the future. Both suggested classification and the CIME technique can improve conservative surgical treatment of women, preserving their uterus and reproductive function and saving the quality of their lives.

#### Duplicate publication statement

The new technique of CIME in this study was based on our previously developed method of intracapsular myomectomy for open and laparoscopic approaches for treating uterine body fibroids (56–58). Therefore, in describing the surgical technique, we have repeated analogous details from the surgical protocols in the previously published articles since the main idea of intracapsular myomectomy is identical in both types of myomectomies. In this study, we adapted the performance of this technique regarding fibroid localization in the cervix and their direct approach via the vagina. There was a publication by Tinelli (59) entitled “Intracervical fibroid removal: a myomectomy reasoned on biological bases,” where the previously published surgical technique of intracervical intracapsular myomectomy through laparoscopy (31) was commented.

## Data Availability

The original contributions presented in the study are included in the article/Supplementary material, further inquiries can be directed to the corresponding authors.

## Footnotes

^1^ https://clincalc.com/stats/power.aspx
