# Supplementary material for: Cervical fibroids: the vaginal intracapsular myomectomy with classification by the fibroids’ origin, growth directions, and localizations
Source: Front Med (Lausanne). 2025 May 9;12:1564667. doi: 10.3389/fmed.2025.1564667 (PMC12101086; doi:10.3389/fmed.2025.1564667)
Supplement: Supplementary file 2 [file Table_2.pdf]

Supplementary Table 2. Retrospective cohort observational study of 32 patients with cervical fibroid removal via the direct vaginal approach.

PTN	Patients' general conditions				Cervical Fibroid's				Surgery			HSD	Hb (g/dL)		SR	RC	FR	PTP		PSMC		CVI
	Age	BMI	PNP	TOD	Symptoms	NBF	SZ	LOC	TM	BL	BT		1st	2nd				PNR	PRO	VG	CX	
1		26	G2P1	TVD	VD, LBP	1	8	ECA	45	85	0	1	13	10,4	1	0	0	0	0	0	0	0
2		24,8	G1P1	TVD	DSP, DSM	1	9	ECA	50	85	0	1	12	10,3	1	1	0	1	TVD	1	0	0
3		26,9	G0P0	-	PP, LAP	1	6,5	ECA	30	50	0	2	10	8,9	1	1, 2	0	0	-	0	0	1
4		27,2	G3P2	TVD	DSP, PP	1	9	ECA	50	95	0	1	13	11,8	1	0	0	0	-	1	0	0
5		21,8	G1P1	TVD	DSM	1	8,5	ECA	45	80	0	1	12	11,3	1	0	0	1	TVD	0	0	0
6		24,1	G0P0	-	PP, LAP	1	6	ECA	35	55	1	2	10	8,4	1	1	0	0	-	1	0	1
7		25,2	G2P1	CS	VD, PP	1	7	ECA	40	80	1	2	12	9,4	1	1	0	0	-	1	1	0
8		28	G3P1	CS	VD, DSP	1	8,5	ECP	45	75	0	1	13	11,2	1	0	0	0	-	0	0	0
9		24	G0P0	-	VD, DCZ	1	8,5	ECP	50	75	0	1	14.1	12.7	1	0	0	1	PTVD	1	0	0
10		22,9	G0P0	-	DSP,VD, DCZ	1	9	ECP	60	80	0	1	13.8	12.3	1	0	1	0	-	0	0	0
11		21,4	G2P1	TVD	VD, LBP	1	8,5	ECP	50	85	0	1	13	11,2	1	0	0	0	-	0	0	1
12		22,8	G3P1	TVD	DSP, DSM	1	8,5	ECP	55	80	0	1	14	12,2	1	0	1	0	-	0	0	0
13		24,8	G1P1	CS	DSP, PP	1	7,5	ECL	25	50	0	1	13	11,2	1	0	0	0	-	0	0	0
14		22,3	G0P0	-	VD, DCZ	1	7,5	ECL	30	65	0	1	13	12,8	1	0	0	1	ABN1	0	0	0
15		26,5	G1P1	TVD	DSP, AUB	1	8,5	ECL	45	70	0	1	12	11,6	1	0	0	0	-	0	0	0
16		28,1	G1P1	TVD	DSM, PP	1	8	ECL	50	85	0	1	12	10,5	1	0	0	0	-	0	0	0
17		24,5	G1P1	TVD	AUB	1	8	ECL	45	65	0	1	12	10,6	1	0	0	0	-	0	0	1
18		27,4	G1P1	CS	DSM, PP	1	8,5	ECL	55	80	0	1	12	10,9	1	0	0	0	-	0	0	0
19		24,1	G0P0	-	DSM, DCZ	1	7,5	ECL	35	60	0	1	12	11,4	1	0	0	0	-	0	0	0
20		23,8	G1P1	TVD	VD, DSM	1	9	ECL	60	80	0	1	12	9,9	1	1	0	0	-	0	1	1
21		25,2	G1P0	AB	VD, DSP	1	6,5	ICL	20	55	0	1	11	9,7	1	1	0	0	-	0	0	1
22		22,6	G1P1	CS	VD, DSP	1	7,5	ICL	35	50	0	1	12	9,9	1	1	0	0	-	0	1	0
23		24,8	G1P0	AB	VD, DSP	1	6	ICL	30	50	0	1	12	9,8	1	1	0	1	TVD	1	0	0
24		30,2	G1P1	TVD	VD, PP	1	6,5	ICL	40	65	0	1	13	11,9	1	0	0	1	TVD	0	0	0
25		22,9	G1P1	CS	DSP, PP	1	8	ICL	50	70	0	1	13	11,3	1	0	0	1	ABN2	1	0	0
26		29,2	G3P2	TVD	AUB, DSP	**2**	**4-7**	ECA,ICP	40	85	0	1	13	11,2	1	0	0	0	-	0	1	0
27		27,1	G3P2	TVD	AUB, DSP	**2**	**5-6**	ECA,ICP	45	80	0	1	13	11,9	1	0	0	0	-	0	0	0
28		26,3	G3P2	TVD,CS	AUB, DSP	**2**	**4,5-8**	ECA,ICP	45	85	0	1	12	11	1	0	0	0	-	0	0	0
29		25,1	G3P3	TVD	DSP, DSM	**2**	**4-6**	ECL,ECL	40	60	0	2	10	9,1	1	1	0	0	-	0	0	1
30		25,1	G3P3	TVD	DSP, DSM	2	4-6	ECL,ECL	60	40	0	1	10,4	9,1	1	1	0	1	TVD	0	0	0
31		23,4	G2P2	TVD	VD, PP	**2**	**5-8**	ECA,ECL	45	75	0	1	13	11,6	1	0	0	0	-	0	1	0
32		32,1	G1P0	AB	DSM, PP	**2**	**4-6**	ECP,ICP	50	70	0	1	12	10,1	1	1	0	0	-	0	1	1

Notes: PTN-patients' number; BMI-body mass index; PNP-parity (nulliparous frequency); TOD-type of delivery; NBF-number of fibroids; SZ-size; LOC-locations; TM-time minutes; BL-blood loss in mL; HBT-blood transfusion; HSD -hospital stay days; Hemoglobin count (Hb) 1st before surgery; Hb 2nd after surgery; SR-cervical fibroids' symptoms resolution; RC-recovery course (0-regular/1-anemia/2-fever); FR-fibroids' recurrence at follow up; PTP-post treatment pregnancies; PNR-number of pregnancies; ABN1-abortion at 9 weeks; ABN2-abortion at 7 weeks; TVD-term vaginal delivery at 39-40 weeks; PTVD-preterm vaginal delivery at 37 weeks; PSMC-postsurgical minor complications, located in vagina (VG) or in cervix (CX); CVI-cervical or vaginal inflammations during follow up; DSP-dyspareunia; PP-pelvic pain; VD-vaginal discharge; LBP-lower back pain; DSM-dysmenorrhea; DCZ-dyschezia; AUB-abnormal uterine bleeding; LAP-lower abdominal pain; CS-Cesarean section; AB-abortion; ECL – extracervical lateral; ECA – extracervical anterior: ECP – extracervical posterior; ICL – intracervical lateral, ICP – intracervical posterior.
