# Supplementary material for: Cervical fibroids: the vaginal intracapsular myomectomy with classification by the fibroids’ origin, growth directions, and localizations
Source: Front Med (Lausanne). 2025 May 9;12:1564667. doi: 10.3389/fmed.2025.1564667 (PMC12101086; doi:10.3389/fmed.2025.1564667)
Supplement: Supplementary file 3 [file Table_3.pdf]

Supplementary Table 3. Studies aimed cervical myomectomy in different populations worldwide with ages of patients and the main perioperative issues. Calculated row parameters are presented as the mean ± standard deviations and median with the lower/upper limits and original variables from the articles included as median with the interquartile (25-75%) or ranges.

N	Studies, n of patients	SA	Study population			Fibroid size, cm	Surgery time, min	Blood loss, ml	Blood loss prevention	Discharge day
			Age, years	NP, %	PCQs					
1	STab. 2: Current study (n=32)	Vag	37.22±3.77<br>37 (31-44)	0.25	WSCFs	7.01±1.59<br>7.5 (4-9) (n=39)	43.75±10<br>45 (20-60)	70.78±13.86<br>75 (50-95)	VPI	1.12±0.34<br>1 (1-2)
2	STab. 6: VMEGP (n=29)	Vag	35.86±10.1<br>37.5 (17-54)	0.26 (n=28)	WSCFs	9.03±6.02<br>6.2 (1-25) (n=54)	NA	136±107.1<br>100 (30-250) (n=5)	VPI[5]	3.13±2.9<br>(1-12) (n=8)
3	STab. 7: VMEOP (n=15)	Vag	28.58±5.47<br>29 (20-37) (n=11)	0.57 (n=8)	ObstCom	7.23±3.48<br>6.6 (4-38) (n=24	NA	265±293.98 (n=3)	VPI	4.75±4.93<br>3 (1-12)<br>(n=4)
4	STab. 8: LSME (n=10)	LS	29.11±6.81<br>30 (18-40)	0.70	WSCFs<br>WWFP	8.1±3.83<br>7 (5-15)<br>(n=16)	175.0±59.16<br>155 (130-260) (n=5)	155±102.14<br>125 (70-300) (n=4)<br>p=0.0312	VPI, VPI +UAE, UAC, TEVBO-BL-IIA, Spon.TM	4.5±4.32<br>2.5 (1-10)<br>(n=5)
5	STab. 9: LTME (n=25)	LT	30.52±7.37<br>30 (17-47)	0.60		12.5±5.78<br>(n=43)	106±36.77<br>106 (80-132) (n=2)			5.82±3.68<br>(n=11)
6	STab. 9: LTGP: Subgroup 1. (n=21)	LT	31.10±7.65<br>30 (17-47)	0.67	WSCFs	12.98±5.98<br>(4.5-30)<br>(n=37)	106±36.77<br>106 (80-132) (n=2)	474.29±602.88<br>300 (50-1800) (n=7, p=0.0045)	VPI	5.87±4.39<br>5.5 (1-15) (n=8)
7	STab. 9: LTCS: Subgroup 2. (n=4)	CS	27.5±5.54<br>28.5 (21-32)	0.25		9.5±3.22<br>10 (6.5-13.5)<br>(n=6)	NA	1533.33±986.58<br>2000 (400-2200) (n=3)	VPI (n=1), Oxytocin	5.67±0.58<br>6 (5-6)<br>(n=3)
8	Wang et al. [31] (n=12)	LS	30.17±4.78<br>29.5 (22-40)	0.50	WWFP	8.65±1.0<br>8.35 (7-10)	106.58±18.21<br>101.5 (82-144)	50±9.54<br>52.5 (30-60)	VPI, Self-made Roeder knot	4.33±0.78<br>4.5 (3-5)
9	Chang et al. [23] (n=28)	LS	38.0±7.0<br>(24-52)	0.57	WSCFs	NA	121.0±56.0<br>121 (45-280)	99.0±114.0<br>(50-500)	GnRHa, BL-UAL, VPI	2.2 (1-5)
10	Higuchi et al. [34] (n=7)	LS	35.5±5.32<br>35 (30-44)	All NP	WWFP	6.6±1.51<br>6.6 (4.9-8.8)	176.43±46.16<br>170 (125-260)	30<br>30 (30-30) (n=5)	GnRHa, VPI,	Within 4 days
11	Lee et al. [36] (n=65)	LS	39.2±6.03<br>38.11(27.40-53.10)	NA	WSCFs	7.3±2.03<br>7.24 (7.18-7.3)	63.25±20.34<br>53.18 (43.12-63.2)	NA	GnRHa, VPI	3
12	Matsuoka et al. [38] (n=16)	LS	37.3±4.2<br>38 (30-41)	0.75	WSCFs	6.56±1.75<br>6 (4-10)	105.81±43.2<br>105.8 (82.8-128.8)	105±117.03<br>105 (42.6-167.4)	GnRHa, VPI	3 (3-3)
13	Sinha et al. [39] (n=24)	LS	36.79±4.52<br>37 (28-45)	All parous	WSCFs	7.87±1.92<br>7.5 (5-11)	88.13±21.25<br>90 (60-120)	79.09±31.31<br>(n=22)		NA

14	Subgroup 1: Sinha et al. [39] (n=12) with UAL	LS	36.33±5.38<br>37.5 (28-43)	All parous	WSCFs	8.5±1.62<br>8.5 (6-11)	91.67±20.38	65.00±24.31	BL-UAL (n=12)	NA
15	Subgroup 2: Sinha et al. [39] (n=12) without	LS	37.25±3.65<br>37 (33-45)	All parous	WSCFs	7.25±2.1<br>7.25 (5-11)	84.58±22.41	96.00±31.34	Without BL-UAL (n=10)	NA
16	Takeuchi et al. [41] (n=5)	LS	36.2±5.3	0.80	WWFP	5.8±1.6	70±18.3	18±8.4	GnRHa, UAC, VPI,	3 (3-3)
17	Zhang et al. [40] (n=13)	LS	38.14±4.43<br>38 (29-46)	0.15	WSCFs	6.63±1.51<br>6.5 (4.2-9.2)	78.46±12.25<br>78 (60-105)	54.23±11.87<br>55 (30-80)	VPI	NA
18	Kaneda et al. [25] (n=10)	LT	35.5±3<br>(28-40)	NA	PWPCFs	Large CFs	157.5±23<br>157.5 (126-218)	727.5±470.5<br>727.5 (80-1962)	IIABOC	NA
19	Tian & Hu [27] (n=9)[¥]	CS	32.8±4.1<br>(25-40)	NA	ObstCom	10.4±7.2<br>(3-30)	NA	697±394<br>(350-4200)	Oxytocin	7.9 (4-20)
20	STab. 10, a: Ch.VME (n=282)	Vag	37.59±2.36	0.31<br>(n=36)	WSCFs	5.26±1.02<br>(n=194)	67.09±14.31	140.06±113.79<br>(n=283)	NA	5.02±0.75<br>(n=272)
21	STab. 10, b: Ch.LSME (n=431)	LS	35.56±2.56<br>(n=427)	0.28<br>(n=130)	WSCFs	6.53±1.87<br>(n=312)	73.77±13.73	112.66±41.17<br>(n=409)	PTI, PTI+VPI	5.7±1.25<br>(n=341)
22	STab. 10, c: Ch.LTME (n=191)	LT	37.52±2.23<br>(n=182)	0.19<br>(n=48)	WSCFs	6.24±1.93<br>(n=84)	81.6±17.27<br>(n=191)	173.69±93.32<br>(n=155)	PTI, PTI+VPI	7.95±1.24<br>(n=115)
23	Dou & Zhang, 2022 [24], (n=7)[¥]	LS	38 (24-52)	NA	WSCFs	7.1 (5-12)	96 (72-135)	75 (10-300)	TPI, water causing method	3 (1-6)
24	Hsiao et al. [35] (n=8)[¥]	LS	47 (43-48)	NA	WSCFs	6.3 (5.5-7.4)	210 (113-293)	175 (100-550)	VPI	4 (3-4)
25	Dou & Zhang [24] (n=11)[¥]	RAL	35 (23-48)	NA	WSCFs	7.9 (5-13)	72 (46-105)	45 (5-200)	TPI	2 (1-5)
26	Hsiao et al. [35] (n=6)[¥]	RAL	48 (44-48)	NA	WSCFs	6.4 (5.2-7.5)	145 (113-195)	200 (96-375)	VPI	4 (4-4)

Notes: STab. – supplementary tables; VMEGyn case report study of vaginal myomectomy in gynecological patients (STab.6); VMEOP – case report study of vaginal myomectomy in obstetric patients (Stab.7); LSME – case report study of laparoscopic myomectomy (Stab.8); LTME – case report study of laparotomic myomectomy (Stab.9), divided into two subgroups: LT in gynecologic patients (LTGP, Stab.9a), myomectomy during Cesarean sections (LTCS, Stab.9b); Chinese studies Ch.VME (Stab.10, a); Ch.LSME (STab.10, b);Ch.LTME (Stab.10, c); NP-nulliparous; LLT loop ligation technique; PCQ – patients' common queries; CFs – cervical fibroids; [¥]cervical myomectomy cases selected among patients excluding those with uterine body myomectomies; MSC – miscellaneous; WSCFs – women with symptomatic CFs; WWFP-women willing fertility preservation; ObstCom.-obstetric complications; PWPCFs – patients with pelvic cavity filled CFs; Gonadotropin-releasing hormone agonists (GnRHa); Vag – vaginal; CS – Cesarean section; LS– laparoscopy; RAL – robot assisted laparoscopy; LT – laparotomy; VPI – vasopressin injection; TPI – terlipressin injection; ENI – epinephrine injection; GnRH a - gonadotropin-releasing hormone agonist; IIABOC – internal iliac artery balloon occlusion; UAC – Uterine artery clipping (clamp); BL-UAL –bilateral uterine artery ligation at its origin; SponTM – SpongostanTM.
