# Supplementary material for: Cervical fibroids: the vaginal intracapsular myomectomy with classification by the fibroids’ origin, growth directions, and localizations
Source: Front Med (Lausanne). 2025 May 9;12:1564667. doi: 10.3389/fmed.2025.1564667 (PMC12101086; doi:10.3389/fmed.2025.1564667)
Supplement: Supplementary file 4 [file Table_4.pdf]

Supplementary Table 4. P-values between comparisons of current study results with data from case report studies and literature.

Compared studies	P values of different parameters and number of patients and samples†:						Statistic tests
	Age (n=32)	NPs (n=32)	CFs' size, (n=39)†	ST (n=32)	BLL (n=32)	HSD (n=32)	
1. STab. 6: VMEGP (n=29)	[a]P=NS (n=27)†	[d]P=NS (n=26)†	[b]P>NS (n=53)†	NA	[b]P=NS (n=5)†	[d]P<0.001 (n=7)†	a). Brown-Forsythe and Welch ANOVA tests with Dunnett's T3 multiple comparisons test. b). Kruskal-Wallis test with Dunn's multiple comparisons test. c). Unpaired two-tailed t-test with Welch's correction for samples with unequal standard deviations. d). Unpaired two-tailed Mann Whitney test.
2. STab. 7: VMEOP (n=15)	[a]P<0.001 (n=13)†	[d]P=0.048 (n=14)†	[b]P>NS (n=25)†	NA	[b]P=NS (n=3)†	[d]P<0.001 (n=4)†	
3. STab. 8: LSME (n=10)	[a]P=0.057 (n=9)†	[d]P=0.02	[b]P=NS (n=16)†	[b]P<0.001 (n=5)†	[b]P=NS (n=4)†	[d]P=0.007 (n=6)†	
4. STab.9: LTME (n=25)	[a]P<0.001	[d]P=0.013	[b]P<0.001 (n=43)†	NA	[b]P<0.001 (n=10)†	[d]P<0.001 (n=11)†	
5. a: LTGP (n=21)	[a]P=0.018	[d]P<0.004	[b]P<0.001 (n=36)†	NA	[b]P=NS (n=6)†	[d]P<0.001 (n=8)†	
6. b: LTCS. (n=4)	[b]P<0.01	[d]P=NS	[b]P>NS	NA	[b]P=0.019	[d]P<0.001 (n=3)†	
7. Higuchi et al.[34] (n=7)	[b]P>NS	NA	[b]P>NS	[b]P<0.001	[b]P=NS	NA	
8. Matsuoka et al. [38] (n=16)	[b]P>NS	[d]P<0.001	[b]P=NS	[b]P<0.001	[b]P=NS	NA	
9. Sinha et al. [39] (n=24)	[a]P>NS	NA	[b]P=NS	[a]P<0.001	[b]P=NS	NA	
10. Subgroup 1: Sinha et al. [39] (n=12) UAL	[a]P>NS	NA	[b]P=0.076	[a]P=NS	[b]P=NS	NA	
11. Subgroup 2: Sinha et al. [39] (n=12) without	[a]P=NS	NA	[b]P=NS	[a]P=0.001	[b]P=NS	NA	
12. Wang et al. [31] (n=12)	[a]P=0.002	[d]P=NS	[b]P<0.001	[a]P<0.001	[b]P<0.001	NA	
13. Zhang et al. [40] (n=13)	[a]P=NS	[d]P=NS	[b]P=NS	[a]P<0.001	[b]P=0.002	NA	
14. Chang et al. [23] (n=28)	[c]P=NS	[d]P=0.0173	NA	[c]P<0.001	[c]P=NS	NA	
15. Takeuchi et al. [41] (n=5)	[c]P=NS	[d]P=NS	[c]P=NS	[c]P=NS	[c]P<0.001	NA	
16. Lee et al. [36] (n=65)	[c]P=0.051	NA	[c]P=NS	[c]P=0.007	[c]P<0.001	NA	
17. Kaneda et al. [25] (n=10)	[c]P=NS	NA	NA	[c]P<0.001	[c]P<0.001	NA	
18. Tian & Hu [27] (n=9)	[c]P=0.013	NA	[c]P=NS	NA	[c]P<0.001	NA	
19. STab. 10, Ch.VME (n=282)	[c]P=NS (n=278)†	[d]P=NS (n=11)†	[c]P<0.001 (n=194)†	[c]P<0.001 (n=194)†	[c]P<0.001 (n=155)†	NA	
20. STab. 10, Ch.LSME (n=431)	[c]P=0.019 (n=427)†	[d]P=NS (n=130)†	[c]P=0.087 (n=312)†	[c]P<0.001 (n=312)†	[c]P<0.001 (n=409)†	NA	
21. STab. 10, Ch.LTME (n=191)	[c]P=NS (n=182)†	[d]P=NS (n=48)†	[c]P=0.022 (n=84)†	[c]P<0.001 (n=84)†	[c]P<0.001 (n=155)†	NA	

Notes: STab – supplementary tables; CFs – cervical fibroids; VMEGP – case report study of vaginal myomectomy in gynecological patients (STab.6); VMEOP – case report study of vaginal myomectomy in obstetric patients (STab.7); LSME – case report study of laparoscopic myomectomy (STab.8); LTME – case report study of open myomectomy (STab.9), divided into two subgroups: LTGP – open myomectomy in gynecologic patients (STab.9a); LTCS – myomectomy during Cesarean sections (STab.9b); Chinese studies: Ch.VME – vaginal (STab.10, a); Ch.LSME – laparoscopic (STab.10, b); Ch.LTME – open myomectomy (STab.10, c); NPs – nulliparous; ST – surgery time; BLL – blood loss; HSD – hospital stay days; UAL – uterine artery ligation; NA – not applicable; † the number of analyzed samples is different from the number of patients in the compared studies.
