# Supplementary material for: Cervical fibroids: the vaginal intracapsular myomectomy with classification by the fibroids’ origin, growth directions, and localizations
Source: Front Med (Lausanne). 2025 May 9;12:1564667. doi: 10.3389/fmed.2025.1564667 (PMC12101086; doi:10.3389/fmed.2025.1564667)
Supplement: Supplementary file 5 [file Table_5.pdf]

Supplementary Table 5. Parallels of the attached sites, growth directions and the depth of cervical fibroids (CFs) with the patients' complaints. These CFs' characteristics and patients' complaints were not registered in all cases in each study

SA	Studies	N	Description of cervical fibroids' manifestations			Clinical manifestation of CFs: patients' complaints
			Attached sites	Growth directions	Growth depth	
Vaginal myomectomy	Suppl. Tab. 2: Current study	15	Ant; Post; Lat	ExtC; IntC; Comb	Within the cervix.	Dyspareunia, pelvic pain, lower back pain, lower abdominal pain, dysmenorrhea, abnormal uterine bleeding, vaginal discharge, dyschezia.
	Suppl. Tab. 6: 27 case reports	29	Ant; Post; Lat; Cent	IntC;; vaginal	The mass filling the vagina; protruding from the vagina prolapsed and hanging mass.	Something was hanging out of the vagina, difficulties of walking, difficulty in micturition, abdominal pain, vaginal discharge with smelling, heavy menstrual and intermenstrual bleeding, dysmenorrhea.
	Suppl. Tab. 7: 15 case reports	15	Ant; Post; Cent	IntC; vaginal	The mass filling the vagina; protruding from the vagina prolapsed and hanging mass.	Vaginal discharge and bleeding during early pregnancy or hemorrhage in advanced pregnancy, lower abdominal pain, a mass extrusion in the vaginal orifice with smelly discharge in postpartum period.
	Suppl. Tab. 10, a: Chinese data: 10 studies	282	Ant; Post; Lat	NA	Subserosal; submucosal; intramural; intramyometrium.	Something was hanging out of the vagina, abdominal fullness or pressure symptom or lump (mass) in the abdomen, urinary urgency or frequency, lower abdominal / pelvic pain, menorrhagia, irregular menses, heavy menstrual bleeding, vaginal bleeding and discharge.
Laparoscopic myomectomy	Suppl. Tab. 8: 10 case reports	10	Ant; Post; Lat	PelC; RetroC; IntraC; PTTV	Pelvic cavity, retrocervical, protruded to the vagina.	Pelvic pressure, urinary frequency, pelvic pain, severe pain during intercourse, chronic fatigue, deep dyspareunia causing severe distress obstructing routine activities, constant menstrual-type cramping, severe heavy menstrual bleeding, menometrorragia, vaginal discharge, infertility.
	Wang et al, 2021 [31]	12	Cent	IntC	Submucosal	NA
	Chang et al., 2010 [23]	28	Ant; Post; Cent; Lat; DRCM	CIMLs; LEIBL; LPIV	Subserosal and intramural lesions; part of lateral lesion extending into the broad ligament; deep rooted cervical myoma or lesion within the	Tenesmus, urinary symptoms, dysmenorrhea, hypermenorrhea, miscarriages, infertility.

					cervix protruding into the vagina.	
	Higuchi et al., 2012 [34]	7	Ant; Post; Lat	NA	Subserosal; submucosal; intramural.	Lower abdominal pain, hypermenorrhea, infertility.
	Lee et al., 2012 [36]	65	Ant; Post; Lat	NA	Subserosal; intramural.	Pressure symptoms (increase in myoma size, palpable mass in abdomen), urinary frequency, constipation, abdominal pain, menorrhagia, dysmenorrhea.
	Matsuoka et al., 2010[38]	16	Cervix	ExtC; IntC	Subserosal; submucosal; intramural; intramyometrium.	Pressure symptoms of the lower abdomen, low back pain, hypermenorrhea, infertility.
	Sinha et al., 2009 [39]	24	Ant; Post	NA	NA	Lump in the abdomen, menorrhagia.
	Zhang et al., 2022 [40]	13	Post	NA	NA	Abdominal fullness, menorrhagia, nonspecific.
	Takeuchi et al., 2006 [41]	5	Cervix	NA	Within the cervix.	Symptoms of the large CFs, the mass protruded into the vagina, anemia due to hypermenorrhea.
	Dou & Zhang, 2022 [24]	18	Cervix	NA	NA	Urinary incontinence, constipation, abdominal pain, menorrhagia, miscarriages / infertility.
	Hsiao et al, 2013 [35]	14	Cervix	NA	NA	Symptoms of large CFs.
	Suppl. Tab. 10, b: Chinese data: 12 studies	431	Ant; Post; Lat	NA	Subserosal; submucosal; intramural; intramyometrium.	Pressure symptom, abdominal fullness or lump in the abdomen, constipation, urinary urgency or frequency, lower abdominal /pelvic pain, menorrhagia, irregular menses, heavy menstrual bleeding, vaginal bleeding, vaginal and discharge.
Laparotomic myomectomy	Suppl. Tab. 9: 25 case reports		Ant; Post	ExtC; IntC; istmC; Comb	CFs filling the pelvic cavity and lower part of the abdomen.	The mass coming out of the vagina, pressure symptoms (abdominal swelling, heaviness in lower abdomen, growing lump in abdomen, increasing abdominal girth), frequent urination intermittent urinary retention and urgency, lower abdominal/pelvic pain, dyspareunia, dysmenorrhea,

						menorrhagia, heavy irregular menstrual bleeding, miscarriages/infertility, vaginal discharge.
	Kaneda et al, 2017 [25]	10	Cervix	Pelvic cavity	Large CFs filling the pelvic cavity.	Symptoms of large CFs.
	Tian & Hu, 2012 [27]	9	Cervix	CR-LUS	CFs filling the pelvic cavity.	Obstetric complications (bleeding).
	Suppl. Tab. 10, c: Chinese data: 7 studies	191	Ant; Post; Lat	NA	Subserosal; submucosal; intramural; Intramyometrium.	Something was hanging out of the vagina, abdominal fullness or pressure symptom or lump (mass) in the abdomen, urinary urgency or frequency, lower abdominal / pelvic pain, vaginal discharge, vaginal bleeding, menorrhagia, irregular menses, heavy menstrual bleeding.

Notes: SA – surgical approaches; App.#2 – case report study of vaginal myomectomy (VME) in gynecological patients; App.#3 – case report study of vaginal myomectomy in obstetric population; App.#4 – case report study of laparoscopic myomectomy; CR#5 – case report study of laparotomic myomectomy (App.a5a VME in gynecologic patients, App.#5b VME during Cesarean sections); App.#6 , Chinese studies (App.#6a VME; App.#6b LSME; App.#6c LTME); CFs – cervical fibroids; NA – not applicable; Ant- anterior; Post – posterior; Lat – lateral; Cent – central; IntCv – intracervical; DRCM – deep rooted cervical myoma; ExtC – extracervical; IntC – intracervical; Comb – combined; PelC - pelvic cavity; RetroC - retrocervical, PTTV - protruded to the vagina; SSLs – subserosal lesions; CIMLs – central intramural lesion; LEIBL – lesion extending into the broad ligament; LPIV – esion within the cervix protruding into the vagina; ExtC; IntC; istmC – istmocervical; Comb ExtC; IntC; istmC – istmocervical; CR-LUS – cervical region of the lower uterine segment.
