# Supplementary material for: Cervical fibroids: the vaginal intracapsular myomectomy with classification by the fibroids’ origin, growth directions, and localizations
Source: Front Med (Lausanne). 2025 May 9;12:1564667. doi: 10.3389/fmed.2025.1564667 (PMC12101086; doi:10.3389/fmed.2025.1564667)
Supplement: Supplementary file 6 [file Table_6.pdf]

Supplementary Table 6. Case reports study. Cervical fibroids' characteristics, perioperative data and age of patients experienced vaginal myomectomy extracted from 29 cases (English language literature).

References		n	Age	NLP	Prot	In vag	Prol. Hang	Ant	Post	Lat	Cent	BLP	Fibroids' size		IOBL, ml	AdPr	DD
													a	b			
Alebrahim et al., 2024		1	43	0				1				VPI	5.2	4.7	50	HS	1
Almajed et al., 2019		2	40	1	1	1			1				10	7			
Baum & Narinedhat, 2009		3	32	0	1	1	1	1					6	4		HS	
Cissé et al., 2008		4	43	0	1	1	1						8,1	6			
Clark et al., 2022		5	44	0	1	1	1						4			HS	
Garg, 2012	a	6	20	1	1	1		1					7	6			
	b	7	25	0	1	1				1			9	7			
Gupta et al., 2018		8	45	1	1		1	1	1				5	5			
Hardon, 1885		9	25	0	1	1		1					6	5			
Ikechebelu et al., 2012		10	37	0	1	1	1				1		22.4	20.5	100		8
Jaouad et al., 2015		11	38	0			1				1		20	17			3
Kanash et al., 2016		12	38	0	1	1	1						25	20			
Kaur et al, 2002		13	35	0	1	1	1		1				8.6	6.2		HS	4
Khan & Seraphim, 2011		14	47	0	1	1	1						11.9	6.2		HS	
Lasmar et al., 2013		15	46	0									2.5	1		HS	1
Majumdar & Gill, 2012		16	17	1		1							2.5	2.5			
Mishra et al., 2016		17	45	0	1	1	1	1					16	14	30		
Nanda et al., 1998		18	50	0	1	1	1						20	15			
Patient et al., 1996		19	23	0				1					3	2		HS-LrS	
Prasanthi, 2020		20	34	0	1	1			1				10	10	50	LS-Ass	2
Rajshree et al., 2017		21	21	1	1	1		1					5.2	6			
Reza et al., 2022		22	47	0	1	1	1						20	8	250		
Rezai et al., 2017		23	29	0									6	4			
Rim Nam et al., 2005		24	54		1	1	1						5.5	5.5			
Taingson et al., 2016		25	31	1	1	1	1			1			13	12	250		4
Torpin et al., 1939		26	29	0	1	1	1		1				15	12			
Wang et al., 2022		27	25	1	1	1	1						8.6	6.2		HS	
Ejikem et al., 2020		28	41	0	1	1		1					4	3.5			2
Sims et al., 1994		29										GnRHa					

Notes: NLP - nulliparous; Prot - protruded; In vag - in vagina; Prol - prolapsed; Hang - hanging; ; Ant - anterior; Post - posterior; Lat - lateral; Cent - central; BLP - blood loss prevention; VPI-vasopressin injection; GnRHa - Gonadotropin-releasing hormone agonists; IOBL - intraoperative blood loss; AdPr - additional procedure (HS - hysteroscopy; LrS-laser surgery; LS-Ass - laparoscopic assistance); DD - discharge day.

References of case reports' study (App.#2).

1. Alebrahim Y, Lim ET, Manias T, Tsampras N. Vaginal myomectomy for a large intrastromal cervical fibroid: a case report. J Surg Case Rep. 2024 Dec 17;2024(12):rjae797. doi: 10.1093/jscr/rjae797. PMID: 39691810; PMCID: PMC11651146.
2. Almajed HI, Radhi HA, Aljufairi Z.Virgin female with undetected cervical fibroid. Bahrain Medical Bulletin 2019; 41(1): 42-44.
3. Baum JD, Narinedhat R. Cervical Myoma Experienced as Prolapse. Journal of Minimally Invasive Gynecology 2009;16:248-9. https://doi.org/10.1016/j.jmig.2008.09.616.
4. Cissé M, Konaté I, Ka O, Dieng M, Dia A, Touré CT. Trachélocèle incarcérée à la suite d'un fibrome géant du col utérin: à propos d'un cas [Incarcerated procidentia due to giant cervical fibromyoma: a case report]. J Gynecol Obstet Biol Reprod (Paris). 2008 Dec;37(8):802-3. French. doi: 10.1016/j.jgyn.2008.09.010. Epub 2008 Nov 1. PMID: 18977609.
5. Clark M, Thigpen B, Sunkara S,Koythong T, Nassif J. Technical Challenges in Hysteroscopic Myomectomy with Prolapsing Cervical Fibroid. JMIG 2022 VOLUME 29, ISSUE 11, SUPPLEMENT , S41, NOVEMBER 2022 DOI:https://doi.org/10.1016/j.jmig.2022.09.135
6. Garg R. Two Uncommon Presentation of Cervical Fibroids. People's Journal of Scientific Research 2012; 5(2):36-8.
7. Gupta A, Gupta P, Manaktala U. Varied Clinical Presentations, the Role of Magnetic Resonance Imaging in the Diagnosis, and Successful Management of Cervical Leiomyomas: A Case-Series and Review of Literature. Cureus 2018. https://doi.org/10.7759/cureus.2653.
8. Hardon VO. A Case of Myoma of the Cervix Uteri. South Med Rec. 1885 Nov 20;15(11):401-403. PMID: 36024062; PMCID: PMC9094348.
9. Ikechebelu JI, Eleje GU, Okpala BC, Onyiaorah IV, Umeobika JC, Onyegbule OA, Ejikeme BT. Vaginal myomectomy of a prolapsed gangrenous cervical leiomyoma. Niger J Clin Pract. 2012 Jul-Sep;15(3):358-60. doi: 10.4103/1119-3077.100648. PMID: 22960976.
10. Jaouad K, Youssef B, Hanane R, Driss M, Mohamed D. Huge prolapsed cervical myoma mimicking cystocele. Saudi J Health Sci 2015;4:135‑7.
11. Kanash S, Savita P, Parmar M. A huge pedunculated cervical fibroid polyp with sepsis--a case report, Journal of Evolution of Medical and Dental Sciences, 2016; 5(99): 7305-7306.
12. Kaur AP, Saini AS, Kaur D, Madhulika Dhillon SP. Huge cervical fibroid - An unusual presentation. J Obstets Gynaecol India2002;52;164-5.
13. Khan A, Seraphim A. Prolapse of a large necrotic cervical fibroid, 8. Journal of Obstetrics and Gynecology 2011;31. https://doi.org/10.3109/01443615.2011.590908.
14. Lasmar BP, Lasmar RB, Pillar C. Office hysteroscopic cervical myomectomy. Gynecol Surg 10, 219-221 (2013). https://doi.org/10.1007/s10397-012-0778-9
15. Majumdar A, Gill K. Severe adolescent menorrhagia due to cervical fibroid. J Obstet Gynaecol India. 2012 Oct;62(5):575-6. doi: 10.1007/s13224-012-0135-y. Epub 2012 May 2. PMID: 24082563; PMCID: PMC3526707.
16. Mishra A, Malik S, Agarwal K, Zaheer S, Gautam A.Huge myxoid leiomyoma of cervix presenting as irreducible prolapse-management by vaginal myomectomy.Int J Reprod Contracept Obstet Gynecol 2016;5:2029-31.
17. Nanda S, Sangwan K, Gulati N. Giant cervical polyp. Trop Doct. 1998 Apr;28(2):112-3. doi: 10.1177/004947559802800225. PMID: 9594689.
18. Patient C, Prentice A, Sutton CJ, Smith SK. Myolysis of a cervical fibroid with an Nd:YAG laser. Br J Obstet Gynaecol. 1996 Jun;103(6):584-5. doi: 10.1111/j.1471-0528.1996.tb09812.x. PMID: 8645655.

19. Prasanthi AV. Laparoscopic assisted vaginal myomectomy of a cervical fibroid-A case report. University Journal of Surgery and Surgical Specialities 2020; 6(8):
20. Rajshree K, Nisha T, Soni M, Sivanandini A. Cervical Fibroids with Its Management and Review of Literature: An Original Article. SEAJCRR 2017; 6(1):5-9.
21. Reza H, Andriani NA, Suwantari SA. Cervical Leiomyoma in Pre-Menopausal Woman. Journal of Biomedicine and Translational Research 2022; 8 (3): 145-148.
22. Rezai S, Bue SL, Bahl N, Chadee A, Gottimukkala S, Fishman A et.al A True Paraurethral Leiomyoma, A Case Report and Review of Literature. Obstet Gynecol Int J 2017; 6(5): 00218. DOI: 10.15406/ogij.2017.06.00218
23. Rim Nam H, Jae Huh S, Taik Park C, Kim B, Ahn G. A case of invasive squamous cell carcinoma on the surface of pedunculated cervical leiomyoma presenting an exophytic cervical cancer. Gynecol Oncol. 2005 Apr;97(1):253-5. doi: 10.1016/j.ygyno.2004.12.021. PMID: 15790470.
24. Taingson MC, Adze JA, Bature SB, Amina DM, Caleb M, Amina A. Vaginal myomectomy of a huge prolapsed cervical leiomyoma. Arch Int Surg 2016;6:127-9.
25. Torpin R & Beard BC. Fibromyoma of Uterine Cervix, Pedunculated and Expelled from Vagina. American Journal of Obstetrics and Gynecology 1940;40(3): 490–492. doi:10.1016/s0002-9378(16)40914-2. Wang L, Feng D, Min J, Transvaginal Myomectomy for A Huge Cervical Myoma with Cervical Cyst: A Case Report and Review of the Literature. Clin Onco. 2022; 6(4): 1-5.
26. Wang L, Feng D, Min J, Transvaginal Myomectomy for A Huge Cervical Myoma with Cervical Cyst: A Case Report and Review of the Literature. Clin Onco. 2022; 6(4): 1-5.
27. Sims JA, Brzyski R, Hansen K, Coddington CC 3rd. Use of a gonadotropin releasing hormone agonist before vaginal surgery for cervical leiomyomas. A report of two cases. J Reprod Med. 1994 Aug;39(8):660 - 2. PMID: 7996536.
28. Ejikem ME, Onyemereze C, Oyamienlen CS. Cervical fibroid: A rare case presentation at Abia State University Teaching Hospital (A case report). Journal of Practical Medicine and Medical Science 2020; 1 (1): 1 - 3.
