# Supplementary material for: Cervical fibroids: the vaginal intracapsular myomectomy with classification by the fibroids’ origin, growth directions, and localizations
Source: Front Med (Lausanne). 2025 May 9;12:1564667. doi: 10.3389/fmed.2025.1564667 (PMC12101086; doi:10.3389/fmed.2025.1564667)
Supplement: Supplementary file 7 [file Table_7.pdf]

Supplementary Table 7. Case reports study. Cervical fibroids' characteristics, perioperative data and age of obstetric patients experienced vaginal myomectomy extracted from 15 cases (English language literature).

References	n	Age	NLP	PW or PP-CS	Prot.	InVag.	Prol. Hang	Ant	Post	Lat	Cent	Fibroids' size		ST, min	IOBL, ml	DD
												a	b			
Abitbol & Madison, 1958	1	34	0	PP-CS					1			7				
Aiyer, 1926	2			Adv.Preg	1							10				
Gonzalez et al., 2020	3	33	0	13	1	1			1			5				1
Gupta et al., 2014	4	35	0	34	1	1	1	1				12	12			
Kamra et al., 2013	5	28	1	35	1			1				5.5	4			
Keriakos & Maher, 2013	6	29	1	PP-CS					1			12.9	9.5			
Kilpatrick et al., 2010	7	32	0	15						1		4	3			
Malhotra & Omran, 2021	8	37	1	PP-CS	1		1	1				9	4			3
Nichols & Hayes, 1953	9	20	1	PP-VD					1			10				
Obara et al., 2014	10	31	1	13	1	1			1			6.20	4.30		145	12
Scot & Spence, 1951	11	20	1	18				1				5				
Sengupta et al., 2006	12	29	0	PP-CS	1	1	1	1			1	12	14		600	
Straub et al., 2010	13		0	18					1			4	3		50	
Tigdi & Chan, 2019	14	28	1	18	1	1						6	5			
Zhang et al., 2018	15	22	1	PP-CS			1	1		1		38	6			3

Notes: NLP - nulliparous; PW - pregnancy weeks; PP-CS-postpartum Cesarean section; Prot – protruded; InVag – in vagina; Prol – prolapsed; Hang – hanging; Ant – anterior; Post – posterior; Lat – lateral; Cent – central; IOBL – intraoperative blood loss; AdS – additional surgery; Adv.Preg. – advanced pregnancy; ST - surgery time; IOBL – intraoperative blood loss; AdS – additional surgery; DD – discharge day.

References of case reports' study.

1. Abitbol MM, Madison RL. Cervical fibroids complicating pregnancy; report of three cases. Obstet Gynecol. 1958 Oct;12(4):397-8. PMID: 13590652.
2. Aiyer GS. A Case of Pregnancy Complicated by Fibroma of the Cervix Uteri. Ind Med Gaz. 1926 Jun;61(6):290. PMID: 29011295; PMCID: PMC5232243.
3. González González V, Herráez Moreta A, Mayoral Triana A, Riolobos Sierra L, Cristóbal García I, Izquierdo Méndez N. Prolapsed cervical myoma during pregnancy. Eur J Obstet Gynecol Reprod Biol. 2020 Sep;252:150-154. doi: 10.1016/j.ejogrb.2020.06.039. Epub 2020 Jun 24. PMID: 32619878.
4. Gupta V, Kumari N, Srivastava M, Nanda, A. Intrapartum Vaginal Myomectomy of a Prolapsed Cervical Fibroid. Journal of South Asian Federation of Menopause Societies, July-December 2014;2(2):99-100 2, 99-100 (2014).
5. Kamra HT, Dantkale SS, Birla K, Sakinlawar PW, Narkhede RR. Myxoid leiomyoma of cervix. J Clin Diagn Res. 2013 Dec;7(12):2956-7. doi: 10.7860/JCDR/2013/6171.3805. Epub 2013 Dec 15. PMID: 24551688; PMCID: PMC3919339.
6. Keriakos R, Maher M. Management of Cervical Fibroid during the Reproductive Period. Case Reports in Obstetrics and Gynecology 2013;2013:1-3. https://doi.org/10.1155/2013/984030.
7. Kilpatrick CC, Adler MT, Chohan L. Vaginal Myomectomy in Pregnancy: A Report of Two Cases. Southern Medical Journal 2010;103:1058-60. https://doi.org/10.1097/smj.0b013e3181efb552.
8. Malhotra S, Omran O. Emergency Vaginal myomectomy of cervical leiomyoma in a pregnant woman at term. J Med Case Rep Case Series 2021; 2(2): https://doi.org/10.38207/jmcrcs20210016
9. Nichols DH, Hayes LW Jr. Cervical fibroid in pregnancy and delivery. Obstet Gynecol. 1953 Aug;2(2):180-2. PMID: 13087911.
10. Obara M, Hatakeyama Y, Shimizu Y. Vaginal Myomectomy for Semipedunculated Cervical Myoma during Pregnancy. American Journal of Perinatology Reports [Internet] 2014;4(01):037-40. Available from: http://dx.doi.org/10.1055/s-0034-1370352.
11. Scott RB, Spence JM Jr. Delivering submucous myoma complicating pregnancy. Am J Obstet Gynecol. 1951 Aug;62(2):447-9. doi: 10.1016/0002-9378(51)90544-3. PMID: 14857091.
12. Sengupta S, Reddy K, Pillai M. Prolapsed cervical fibroid in pregnancy: A challenging obstetric dilemma. Journal of Obstetrics and Gynaecology 2006;26:823-4. https://doi.org/10.1080/01443610600994700.
13. Straub HL, Chohan L, Kilpatrick CC. Cervical and Prolapsed Submucosal Leiomyomas Complicating Pregnancy. Obstetrical & Gynecological Survey 2010;65:583-90. https://doi.org/10.1097/ogx.0b013e3181fc5602.
14. Tigdi J, Chan C. A Case Report of Overcoming an Obstructive, Pedunculated Cervical Fibroid at the Time of Uterine Evacuation. Case Reports in Obstetrics and Gynecology 2019 Article ID 2651680, 3 pages https://doi.org/10.1155/2019/2651680
15. Zhang J, Zou B, Wang K. Spontaneous expulsion of a huge cervical leiomyoma from the vagina after cesarean. Medicine 2018;97:e11766. https://doi.org/10.1097/md.0000000000011766.
