# Supplementary material for: Cervical fibroids: the vaginal intracapsular myomectomy with classification by the fibroids’ origin, growth directions, and localizations
Source: Front Med (Lausanne). 2025 May 9;12:1564667. doi: 10.3389/fmed.2025.1564667 (PMC12101086; doi:10.3389/fmed.2025.1564667)
Supplement: Supplementary file 8 [file Table_8.pdf]

Supplementary Table 8. Case reports study. Cervical fibroids' characteristics, perioperative data and age of patients experienced laparoscopic myomectomy extracted from 10 case reports (English language literature).

References	n	Age	NPS	Post.	Fibroids' size		VPI	TBOIIA	ST, min	IOBL, ml	DD	PS Compl
					a	b						
Garzon-Lopez et al., 2015	1	31	0		15		1		260		10	Abs.1
Giannella et al., 2016	2	18	1	1	7						3	
McEntee & Dahlam, 2022	3	34	0		5					100		
Peker et al., 2017	4	40	1	1	14	10			140	300	1	
Simon et al., 2021	5	30	1		12	8			170	150	1	
Sleiman et al., 2023	6	28	1	1	14							
Sunkara et al., 2021	7		1		8	8	1	BUAC				
Takeda et al., 2009	8	33	1	1	6	2.6	1	1	130	70	10	
Wang et al., 2008	9	28	0		5.5	4.5						
Wehbe et al., 2016	10	20	1	1	5	5	1				2	

Notes: NPS-nulliparous; Post – posterior; VPI – vasopressin injection; TBOIIA-temporary balloon occlusion of the bilateral internal iliac arteries; BUAC – bilateral uterine artery clipping; ST-surgery time; IOBL – intraoperative blood loss; DD – discharge day; PSC – postsurgical; complications; Abs – abscess..

References of case reports' study.

1. Garzon-Lopez, O., Garzón-Lopez, F., Gomez-Ponce, H., & Morgan-Ortiz, F. Laparoscopic Management of a Huge Retro-Cervical Myoma. Journal of Minimally Invasive Gynecology, 2015;22(6), S218. https://doi.org/10.1016/j.jmig.2015.08.774.
2. Giannella L, Mfuta K, Tuzio A, Cerami LB. Dyspareunia in a Teenager Reveals a Rare Occurrence: Retroperitoneal Cervical Leiomyoma of the Left Pararectal Space. J Pediatr Adolesc Gynecol. 2016;29(1):e9-e11. doi: 10.1016/j.jpag.2015.08.005. Epub 2015 Aug 22. PMID: 26307239.
3. McEntee K, Dahlman M. Laparoscopic myomectomy of a lateral cervical fibroid with temporary uterine artery occlusion. American Journal of Obstetrics and Gynecology [Internet] 2022;226(3):S1365. Available from: http://dx.doi.org/10.1016/j.ajog.2021.12.214.
4. Peker N, Gündoğan S, Şendağ F. Laparoscopic Management of Huge Cervical Myoma. Journal of Minimally Invasive Gynecology 2017;24:345-6. https://doi.org/10.1016/j.jmig.2016.09.002.
5. Simon V. Laparoscopic Cervical Myomectomy with PRE-Operative Uterine Artery Embolization and Concomitant Abdominal Cerclage: A Case Report. Journal of Minimally Invasive Gynecology 2020;27:S98. https://doi.org/10.1016/j.jmig.2020.08.127.
6. Sleiman Z, Ayed A, Christoforou C, Petousis S, Alkatout I, Gitas G, Geru M, Buzzaccarini G. Posterior cervico-vaginal myomectomy: a laparoscopic technique 2023; 35(1):21-5. doi: 10.36129/jog.2022.36
7. Sunkara S, Koythong T, Nijjar JB, Chohan L. Cervical Fibroid Myomectomy. JMIG 2021 VOLUME 28, ISSUE 10.1016/j.jmig.2021.09.332 11, SUPPLEMENT , S22, NOVEMBER 2021
8. Takeda A, Koyama K, Imoto S, Mori M, Sakai K, Nakamura H. Temporary endovascular balloon occlusion of the bilateral internal iliac arteries for control of hemorrhage during laparoscopic-assisted myomectomy in a nulligravida with a large cervical myoma. Fertil Steril. 2009 Mar;91(3):935.e5-9. doi: 10.1016/j.fertnstert.2008.09.040. Epub 2008 Nov 6. PMID: 18990372.
9. Wang KC, Kim JH, Advincula AP. Robot Assisted Laparoscopic Excision of a Cervical Myoma. Journal of Minimally Invasive Gynecology 2008;15:11S. https://doi.org/10.1016/j.jmig.2008.09.039.
10. Wehbe GS, Doughane M, Bitar R, Sleiman Z. Laparoscopic posterior Colpotomy for a Cervico-vaginal Leiomyoma: hymen conservative technique. Facts Views Vis Obgyn. 2016 Sep;8(3):169-171. PMID: 28003871; PMCID: PMC5172573.
