# Supplementary material for: Cervical fibroids: the vaginal intracapsular myomectomy with classification by the fibroids’ origin, growth directions, and localizations
Source: Front Med (Lausanne). 2025 May 9;12:1564667. doi: 10.3389/fmed.2025.1564667 (PMC12101086; doi:10.3389/fmed.2025.1564667)
Supplement: Supplementary file 9 [file Table_9.pdf]

Supplementary Table 9. Case reports study. Cervical fibroids' characteristics, perioperative data and age of patients experienced laparotomic myomectomy extracted from 25 cases (English language literature) of laparotomic gynecological patients (Suppl. Tab. 9, a: 1-21 cases) and obstetric population (Suppl. Tab. 9, b: 22-25 cases).

	References	n	Age	NPS	Ant.	Post.	Lat.	Cent.	GnRHa	VPI	UAE	Fibroids' size		ST, min	IOBL, ml	DD
												a	b			
a: Gynecological patients	Abdelaziz et al., 2014	1	39	0		1						9				
	AbuHasim et al., 2020	2	27	1	1				1			10.5	9	80	150	2
	Akhan et al., 2022	3	40	0		1	1				1	11.5			520	4
	Anant & Gupta, 2020	4	24	0		1				1		20	18			
	Booher et al., 2018	5	33	0						1		4.5	6		50	1
	Eigbefoh et al., 2008	6	34	1		1						14	12		200	7
	Elegbua et al., 2023	7	24	1	1		1			1		7.86	7.09			6
	Galidevara & Rajarajan, 2021	8	29	0				1		1		18	16			
	Gupta et al., 2018a	9	30	1				1				15	13			
	Gupta et al., 2018b	10	30	1	1							18	20			
	Güzin et al., 2005	11	25	1	1							12.50	7			
	Higuchi et al., 2012	12	29	1		1			1	1		12		132	1800	4
	Noguchi et al., 2019	13	37	1								18				
	Palve et al., 2017	14	47	1								13	20		300	
	Peng et al., 2016	15	42	0								7.40	10			
	Rajshree et al., 2017	16	37	1	1							6.6	5.4			
	Sharma et al., 2014	17	22	1	1							30	25			15
	Shradha & Sahay, 2016	18	17	1	1							18	20			8
	Wakode & Lodha, 2022	19	36	0								11	12			
	Yalçin et al., 2016	20	30	1								5	6			
	Yeoh et al., 2019	21	21	1								12			300	
b: Obstetric patients	Chaitra et al., 2017	22	32	0	1							6.50	5		400	5
	Erian et al., 2004	23	25	0								10	10		2000	6
	Gundabattula et al., 2020	24	21	0								13.5	12		2200	6
	Murakami et al., 2007	25	32	1												

Notes: NPS-nulliparous; Ant.-anterior; Post.-posterior; Lat-lateral; Cent-central; GnRHa - Gonadotropin-releasing hormone agonists; VPI – vasopressin injection; TBOIIA-Temporary balloon occlusion of the bilateral internal iliac arteries; ST-surgery time; IOBL – intraoperative blood loss; DD – discharge day.

References of case reports' study (App.#5)

1. Abdelaziz A, Joseph S, Ashraf M, Abuzeid M. Myometrial Abscess: A Complication of Myomectomy of a Large Cervical Myoma. Journal of Minimally Invasive Gynecology [Internet] 2014;21(6):S195-6. Available from: http://dx.doi.org/10.1016/j.jmig.2014.08.632
2. AbuHashim H, Al Khiary M, El Rakhawy M. Laparotomic myomectomy for a huge cervical myoma in a young nulligravida woman: A case report and review of the literature. Int J Reprod Biomed. 2020 Feb 27;18(2):135-144. doi: 10.18502/ijrm.v18i2.6421. PMID: 32259008; PMCID: PMC7097170.
3. Akhan SE, Yasa C, Dural O, Ugurlucan FG, Rozanes I. Successful pregnancy after presurgical uterine artery embolization in the management of a very large cervical myoma: A case report. Case Reports in Women's Health 2022;36:e00450. https://doi.org/10.1016/j.crwh.2022.e00450.
4. Anant M, Gupta S. Cervical Reconstruction after Cervical Myomectomy or Hysterectomy: Operative Challenges in Huge Cervical Fibroids. J South Asian Feder Obst Gynae 2020;12(4):254-257.
5. Booher M, Edelson M, Jaspan D, Goldberg J. Myomectomy of a large cervical fibroid in a patient desiring future fertility. OBG Management 2018;30(10):20-24.
6. Eigbefoh J, Onuminyan D, Abebe J. Intermittent urinary retention secondary to a cervical leiomyoma. Trop J Obstet Gynaecol. 2008;25:112-116. https://www.ajol.info/index.php/tjog/article/view/85020.
7. Elegbua CO, Afolayan ST, Archibong EI, Eze IO. Uterine conserving cervical myomectomy in a young nulliparous woman in Navy Reference Hospital Calabar, Nigeria. Sch Int J Obstet Gynec 2023; 6(5): 174-177.
8. Galidevara C, Rajarajan D. Cervical Fibroid Managed by Uterus Conserving Surgery. Journal of Clinical and Diagnostic Research, 2021;15(2):QD01-QD02. https://www.jcdr.net/articles/PDF/14569/47162_CE[Ra]_F(Sh)_PF1(SK1_KM)_PFA(SHU)_PN(SHU).pdf
9. Gupta A, Gupta P, Manaktala U. Varied Clinical Presentations, the Role of Magnetic Resonance Imaging in the Diagnosis, and Successful Management of Cervical Leiomyomas: A Case-Series and Review of Literature. Cureus 2018. https://doi.org/10.7759/cureus.2653.a
10. Gupta, N., Jahan, U. & Usmani, F. A Huge Cervical Fibroid with Infertility: Case Report. International Journal of Current Research 2010; 18: 73747-73748.b
11. Güzin K, Sezginsoy S, Tuncay YA, Naki M, Kanadikirik F. The management of an unusually sited isthmicocervical leiomyoma and a huge prolapsed pedunculated submucous leiomyoma. Gynecol Surg 2005;2:35-38. https://doi.org/10.1007/s10397-005-0086-8.
12. Higuchi Y, Okuda K, Nakamura Y, Hayashi A, Hayashi M, Fujiyama F, et al. Efficacy and safety of bipolar electrode grasping forceps for laparoscopic myomectomy in uterine cervical myoma. Asian Journal of Endoscopic Surgery 2012;5:126-30. https://doi.org/10.1111/j.1758-5910.2012.00140.x.
13. Noguchi M, Kitajima M, Abe S, Murakami N, Kitajima Y, Miura K, Masuzaki H. Huge uterine fibroid arising from primary uterine cervical diverticulum: a case report and review of the literatures. J Obstet Gynaecol. 2019 Nov;39(8):1186-1187. doi: 10.1080/01443615.2019.1588237. Epub 2019 Jun 18. PMID: 31210084.
14. Palve TT, Katke RD, Saha D. A rare case of huge cervical fibroid. J. Evid. Based Med. Healthc. 2017; 4(55), 3382-3383. DOI: 10.18410/jebmh/2017/672
15. Peng K, Jiang LY, Teng SW, Wang PH. Degenerative leiomyoma of the cervix: Atypical clinical presentation and an unusual finding. Taiwanese Journal of Obstetrics and Gynecology 2016;55(2):293-5. https://doi.org/10.1016/j.tjog.2016.01.001
16. Rajshree K, Nisha T, Soni M, Sivanandini A. Cervical Fibroids with Its Management and Review of Literature: An Original Article. SEAJCRR 2017; 6(1):5-9.
17. Sharma S, Kaur EJ, Thakur R, Kalsi M, Kotwal S. Myomectomy and Cervical Reconstruction in an Unmarried Girl with Large Cervical Fibroid.JK Science Journal of Medical Education and Research. 2014; 16(2): 94-96. http://imsear.searo.who.int/handle/123456789/172409
18. Shradha, Sahay PB. Unusual Presentation of Cervical Fibroid. Indian Journal Of Applied Research 2016; 6(4):202-3.
19. Wakode SR, LodhaRV. Cervical leiomyoma: a rare case with unusual presentation.Int J Reprod Contracept Obstet Gynecol 2022;11:275-8. https://dx.doi.org/10.18203/2320-1770.ijrcog20215120

20. Yalçin I, Pabuçcu E, Kahraman K, Sönmezer M. Mini-laparotomic Colpotomy for a Cervicovaginal Leiomyoma: Preservation of hymenal integrity. Int J Reprod Biomed. 2016 Mar;14(3):217-20. PMID: 27294222; PMCID: PMC4899768.
21. Yeoh S, Simcock B, Sykes P. A surgical approach to management of a large cervical leiomyoma with fertility preservation. ASGO ASM 2019. Sydney 5-9 June 2019 https://static1.squarespace.com/static/5b26fc513917eed567612e10/t/631ec1dc854ced2ee5f56143/1662960132767/ASGO+ASM+2019.pdf
22. Chaithra TM, Leena GP, Soman U, et al. Caesarean myomectomy in a cervical fibroid: a brief communications. Int J Pregn & Chi Birth. 2017;2(1):17-19. DOI: 10.15406/ipcb.2017.02.00009
23. Erian J, El-Toukhy T, Chandakas S, Kazal O, Hill N. Rapidly enlarging cervical fibroids during pregnancy: a case report. J Obstet Gynaecol. 2004 Aug;24(5):578-9. doi: 10.1080/01443610410001722743. PMID: 15369948.
24. Gundabattula SR, Bayyarapu VB, Pochiraju M, Pawar S, Gala AR. Caesarean myomectomy of a large cervical fibroid. Archives of Gynecology and Obstetrics 2020;301:859-61. https://doi.org/10.1007/s00404-020-05446-0.
25. Murakami T, Niikura H, Shima Y, Terada Y, Okamura K. Sloughing off of a cervical myoma after cesarean section: a case report. J Reprod Med. 2007 Oct;52(10):962-4. PMID: 17977176.
