# Supplementary material for: Cervical fibroids: the vaginal intracapsular myomectomy with classification by the fibroids’ origin, growth directions, and localizations
Source: Front Med (Lausanne). 2025 May 9;12:1564667. doi: 10.3389/fmed.2025.1564667 (PMC12101086; doi:10.3389/fmed.2025.1564667)
Supplement: Supplementary file 10 [file Table_10.pdf]

SupplementaryTable 10. Perioperative results of cervical myomectomy depending on type of surgery from Chinese language literature (904 cases).

| SA | Variables (numbers, mean± SD) | References and study results | | | | | | | | | | | | | | | |
|---|---|---|---|---|---|---|---|---|---|---|---|---|---|---|---|---|---|
| | | Du & Wang, 2007 | He et al., 2011 | Jiang. 2007 | Ren et al., 2019 | Song et al., 2012 | Tie & Ge, 2017 | Wang et al., 2014 | Wang, 2018 | Wei, 2016 | Wei, 2020 | Wu, 2019 | Yi & Sun, 2018 | Yin, 2016 | Yu & Luo, 2016 | Zhang et al., 2017 | Total or summarized mean±SD & p values |
| a).VME | n | - | - | 8 | - | 6 | 14 | 36 | 37 | 34 | 65 | - | - | 50 | 4 | 28 | 282 |
| | Age, mean± SD | - | - | 43±11 | - | 33.5 (23-48) | 41.2±2.6 | 34.2±4.8 | 38.3±5.1 | 36.6±5.5 | 38.1±4.1 | - | - | 36.2±2.4 | | 37.5±8.1 | 37.59±2.36 (n=278) (p=NS) |
| | Nulli 5parous, mean± SD | - | - | - | - | - | - | 11 | - | - | - | - | - | - | - | NA | 0.31±0.47 NS (n=11) (p=NS) |
| | Size, cm | - | - | <5 | - | 7.5±3.5 (3-6 to 5-15) | NA | 4.9±2.6 | NA | 6.5±1.4 | 5.1±2.0 | - | - | 3.9±0.9 | NA | 6.2±1.7 | 5.26±1.02 (n=194) (p<0.001) |
| | Surgery time, min | - | - | 68 | - | 26.4 | 59.4±7.4 | 78.4±25.3 | 82.3±19.3 | 83.4±25.8 | 54.4±9.9 | - | - | 72±22 | 67.3 | 45.6±19.3 | 67,09±14,31 (p<0.001) |
| | Blood loss, ml | - | - | 150 | - | 20 | NA | 116.4±40.5 | 111.9±23.6 | 96.9±48.7 | 56.3±6.3 | - | - | 376±31 | 121 | 53.6±18.8 | 139.51±116.92 (n=268) (p=0.0264) |
| | Blood loss prevention | - | - | NA | - | PTI | PTI | PTI | NA | NA | PTI | - | - | NA | PTI | NA | Yes: 5/10 |
| | Discharge day | - | - | 4,8 (n=8) | | NA | 4.5±0.52 | 4.4±1.2 | 4.06±1.22 | 4.35±1.45 | 5.2±1.3 | - | - | 6.2±1.2 | NA | 5.7±1.3 | 5.02±0.75 (n=272) (p<0.001) |
| b).LSME | n | 46 | 48 | - | 37 | - | 22 | 36 | 37 | 33 | 65 | 39 | 47 | - | 4 | 17 | 431 |
| | Age | 31.2±6.09 (21-42) | 35.6±4.5 | - | 37.48±5.62 | - | 41.9±2.6 | 35.1±4.6 | 37.8±5.3 | 36.2±5.4 | 36.8±3.7 | 31.84±1.04 | 34.72±5.69 | - | NA | 35.8±2.3 | 35.56±2.55 ( n=427) (p=0.0183) |

| | | | | | | | | | | | | | | | | | |
|---|---|---|---|---|---|---|---|---|---|---|---|---|---|---|---|---|---|
| | Nulli-parous | 17 (0.37±0.49) | 12 (0.25±0.44) | - | - | - | - | 8 (0.22±0.42) (n=NS) | - | - | - | NA | NA | - | NA | NA | 0.28±0.45 (n=130) (n=NS) |
| | Size, cm | 5.8±2.72 (3-15) | 8.3±2.1 | - | 4.31±1.23 | - | NA | 5.2±2.4 | NA | 6.4±1.3 | 5.3±1.9 | NA | >10 cm | - | NA | NA | 6,53±1.87 (n=312) (p=0,049) |
| | Surgery time, min | 75±45 (25-140) | 91.8±38.5 | - | 59.1±19.6 | - | 63.1±10.7 | 99.5±45.5 | 97.6±15.6 | 85.2±24.6 | 73.7±16.2 | 58.13±4.04 | 62.37±20.54 | - | 96.7 | 62.0±27.1 | 73.77±13.73 (p<0,001) |
| | Blood loss, ml | 45±30 (10-200) | 185.5±88.5 | - | 70.19± 63.42 | - | NA | 150.1±88.5 | 149.7±30.9 | 100.2±47.3 | 117.2±17.1 | 95.34±3.66 | 114±35 | - | 118 | 64.2±25.9 | 112.66±41.17 (n=409) (p<0,001) |
| | Blood loss prevention | PTI | PTI | - | PTI+VPI | - | PTI | PTI | NA | NA | PTI | NA | PTI | - | PTI | None | Yes: 8/12 |
| | Discharge day | 6.4±1.2 | 4.3±0.6 | | 5.88±1.57 | - | 4.41±0.5 | 5.2±1.8 | 5.61±1.46 | 4.21±1.42 | 7.8±1.9 | NA | NA | - | NA | 5.2±1.1 | 5.7±1.25 (n=341) (p<0,001 |
| c).LTME | n | - | 48 | - | 19 | 23 | 36 | - | - | - | - | 39 | - | 17 | 9 | - | 191 |
| | Age, mean± SD | - | 37.2±5.8 | - | 35.74±5.99 | 33.5 (23-48) | 41.1±3.1 | - | - | - | - | 37.67±1.02 | - | 37.9±2.8 | NA | - | 37,52±2.23 (n=182) (p=NS) |
| | Nulliparous, mean± SD | - | 9 | - | - | - | - | - | - | - | - | NA | - | - | NA | - | 0.19±0.39 (n=48 (p=NS) |
| | Size, cm | - | 7.8±2.8 | - | 5.13±2.16 | - | - | - | - | | - | NA | - | 3.1±0.9 | NA | - | 6.24±1.93 (n=84) (p=0,019) |
| | Surgery time, min | - | 99.9±46.6 | - | 48.1±16.75 | 70.5 | 98.8±11.3 | - | - | - | - | 72.21±6.06 | - | 70±29 | 78.6 | - | 81.6±17.27 (p<0,001) |
| | Blood loss, ml | - | 193.8±109.8 | - | 107.17±96.65 | 100.0 | - | - | - | - | - | 126.57±5.38 | - | 418±42 | 138 | - | 173,74±93.44 (n=155) (p<0,001) |

| | | | | | | | | | | | | | | | | | |
|---|---|---|---|---|---|---|---|---|---|---|---|---|---|---|---|---|---|
| | Blood prevention | - | PTI | - | PTI+VPI | PTI | OT | - | - | - | - | NA | - | NA | PTI | - | Yes: 4/6 |
| | Discharge day | | 6.7±1.3 | - | 7.8±1.5 | NA | 8.92±0.91 | - | - | - | - | NA | - | 11.3±1.6 | NA | | 8.19±1.57 (n=120) (p<0,001) |

Notes: SA- surgery approaches; VME – vaginal myomectomy; LSME – laparoscopic myomectomy; LTME – laparoscopic myomectomy; SD – standard deviation; VPI – vasopressin injection; PTI – pituitrin injection; OT- oxytocin injection; NA – no available.

| N | References translated into English | Original references in Chinese |
|---|---|---|
| 1 | Du W, Wang X. Value of laparoscopic myomectomy in women with cervical lelomyoma. China Journal of Endoscopy 2007, 13(7):729-730, 734 | 杜炜杰，汪晓菁。腹腔镜在宫颈肌瘤手术中的应用。中国内镜杂志2007年第13卷第7期729-730, 734页 |
| 2 | He S, Yao S, Zhang C, You Z, Li J. Clinical Study of Laparoscopic Myomectomy. Guangdong Medical Journal 2011,(32(11):1460-1463. | 何善阳，姚书忠，张彩，游泽山，李捷。腹腔镜宫颈肌瘤剔除术的临床研究。广东医学 2011 年第 32 卷 11 期 1460-1463 页 |
| 3 | Jiang S. Surgical treatment for 44 cases of uterine cervicai leiomyoma. Maternal & Child Health Care of China. 2007, 22:226-227 | 蒋仕祥. 44 例宫颈肌瘤手术探究. 中果妇幼保健 2007 年第 22 卷 226-227 页 |
| 4 | Ren Y, Xing X, Zhai Y, Cheng Q, Wang Q. Comparison of two surgical methods for cervical leiomyoma. Capital Food Medicine. 2019, 3: 42-43. | 任亚娟 邢雪姣 翟艳红 成清奇，王清华。宫颈肌瘤的两种手术方式的比较。首都食品与医药 2019 年 3 月下 42-43 页 |
| 5 | Song N, Ma X, Zhang S. Surgical Treatment Experience of 38 Cases of Cervical Myoma. Shanxi Med J, 2012,41(5):492-493. | 宋宁，马晓欣，张淑兰。宫颈肌瘤38例手术治疗体会。山西医药杂志2012年5月第4l卷第5期上半月492-493页 |
| 6 | Tie W, Ge F. Comparative analysis of clinical curative effect of different treatment methods for cervical leiomyoma. Modem Practical Medicine, 2017,29(2):234-235,248. | 铁炜炜，葛芬芬.宫颈肌瘤不同治疗方法临床疗效比较分析.现代实用医学 2017 年 2 月第 29 卷第二期 234-235,248 页 |
| 7 | Wang J, Cheng J, Jin J, Li L. Therapeutic Effects of Transvaginal Cervical Myomectomy and Laparoscopic Cervical Myomectomy. Chin J Obstet Gynecol Pediatr (Electron Ed), 2014,10(6):779-782. | 王金娟，成九梅，金婧，李琳。宫颈肌瘤经阴道剔除术与经腹腔镜剔除术的疗效观察。中华妇幼临床医学杂志(电子版)2014 年 12 月第 10 卷第 6 期779-782页 |
| 8 | Wang J. Comparison of clinical efficacy between vaginal surgery and laparoscopic surgery in the treatment of cervical fibroids. Health Literature, 2018,9:188. | 王靖。阴式手术与腹腔镜手术治疗宫颈肌瘤临床疗效对比。保健文汇 2018 年 9 期 188 页 |
| 9 | Wei X. Clinical analysis of vaginal and laparoscopic excision of cervical myoma. Journal Of Practical Gynecologic Endocrinology, 2016,3(13):39- | 韦 歆。宫颈肌瘤经阴道剔除术 |

| | | |
|---|---|---|
| | 40. | 与经腹腔镜剔除术的临床分析。实用妇科内分泌杂志 2016 年 9 月 C 第 3 卷/第 13 期 39-40 页 |
| 10 | Wei J. Effects of different myomectomy methods on the postoperative body stress state and cervical local microcirculation. J Mod Med Health, 2020,36(8):1198-1201. | 魏菊红。子宫肌瘤剔除术不同术式对患者术后机体综合应激状态及宫颈局部微循环影响研究。现代医药卫生 2020 年 4 月第 36 卷第 8 期1198-1201页 |
| 11 | Wu J. Analysis of the clinical effect of laparoscopic myomectomy on the posterior wall of the cervix. World Latest Medicine Infomation. 2019, 19(39): 68, 71. | 武金花。经腹腔镜子宫颈后壁肌瘤手术切除的临床效果分析。世界最新医学信息文摘 2019 年第 19 卷第 39 期 68, 71 页 |
| 12 | Yi X, Sun J. The Clinical Analysis of 47 Cases of Laparoscopic Large Cervical Myomectomy. Chinese Practical Journal of Rural Doctor. 2018,25(2): 71-72, 75. | 伊喜苓，孙建华。腹腔镜下巨大宫颈肌瘤切除术 47 例临床分析。中国实用乡村医生杂志，2018，25(2）71-72, 75 页 |
| 13 | Yin S. Clinical Analysis of 50 Cases of Cervical Myoma by Transvaginal Hysterectomy. Chinese and Foreign Medical Research. 2016,14(5):52-53 | 尹素梅。经阴道手术切除子宫颈肌瘤 50 例临床分析。中外医学研究第 14 卷 第 5 期 52-53 页 |
| 14 | Yu L, Luo L. Clinical Analysis of Surgical Treatment of 39 Cases of Cervical Myoma. Yinshi Baojian, 2016,3(10):1-2. | 于丽，罗丽。宫颈肌瘤 39 例手术治疗临床分析。饮食保健 2016 年 5 月(下) 第 3 卷第 10 期 1-2 页 |
| 15 | Zhang N, Zhao E, Zhao F, Xu F. Effectiveness of vaginal myomectomy vs laparoscopic myomectomy for cervical myoma. Acad J Chin PLA Med Sch, 2017,38(7):631-633, 638. | 张宁宁，赵恩锋，赵芳，徐锋。阴式手术与腹腔镜手术治疗宫颈肌瘤临床疗效探究。解放军医学院学报 2017， 38(7):631-633, 638 |
