# Supplementary material for: Cervical fibroids: the vaginal intracapsular myomectomy with classification by the fibroids’ origin, growth directions, and localizations
Source: Front Med (Lausanne). 2025 May 9;12:1564667. doi: 10.3389/fmed.2025.1564667 (PMC12101086; doi:10.3389/fmed.2025.1564667)
Supplement: Supplementary file 11 [file Table_11.pdf]

Supplementary Table 11. Post-hoc power analysis and sample size calculation using parameters of current retrospective study (n=32 and nulliparous incidence of 25%) and variables of independent studies from the literature at the alpha (0.05), beta (0.2) and power (0.8) performed online [https://clincalc.com/stats/power.aspx]						
**Studies and number of patients**	**Nulliparous incidence, %**	**P value versus current study**	**Post-hoc power**	**Calculated sample size for groups**	**Calculated total sample size**	**Actual sample size**
**LS myomectomy, case reports (n=10)**	**78**	**0.0202**	**89**	**13**	**26**	**42**
**LT myomectomy, case reports (n=21)**	**63**	**0.0136**	**80.1**	**26**	**52**	**53**
**Matsuoka et al, 2010 [38], (n=16)**	**76**	**0.0016**	**94.8**	**14**	**28**	**48**
**Chang et al., 2010 [23], (n=28)**	**57**	**0.0173**	**72.3**	**36**	**72**	**60**

Clinical Calculators (ClinCalc.com). Post-hoc power calculator. https://clincalc.com/stats/power.aspx
